# Understanding the contribution of lifestyle in breast cancer risk prediction: a systematic review of models applicable to Europe

**DOI:** 10.1186/s12885-023-11174-w

**Published:** 2023-07-21

**Authors:** Elly Mertens, Antonio Barrenechea-Pulache, Diana Sagastume, Maria Salve Vasquez, Stefanie Vandevijvere, José L. Peñalvo

**Affiliations:** 1 Unit of Non-Communicable Diseases, Department of Public Health, Institute of Tropical Medicine, Nationalestraat 155, 2000 Antwerp, Belgium; 2 Health Information, Scientific Institute of Public Health (Sciensano), Brussels, Belgium; 3 Global Health Institute, University of Antwerp, Antwerp, Belgium

**Keywords:** Breast cancer, Risk prediction, Europe, Model performance, Lifestyle, BMI, Alcohol, Physical activity

## Abstract

**Background:**

Breast cancer (BC) is a significant health concern among European women, with the highest prevalence rates among all cancers. Existing BC prediction models account for major risks such as hereditary, hormonal and reproductive factors, but research suggests that adherence to a healthy lifestyle can reduce the risk of developing BC to some extent. Understanding the influence and predictive role of lifestyle variables in current risk prediction models could help identify actionable, modifiable, targets among high-risk population groups.

**Purpose:**

To systematically review population-based BC risk prediction models applicable to European populations and identify lifestyle predictors and their corresponding parameter values for a better understanding of their relative contribution to the prediction of incident BC.

**Methods:**

A systematic review was conducted in PubMed, Embase and Web of Science from January 2000 to August 2021. Risk prediction models were included if (i) developed and/or validated in adult cancer-free women in Europe, (ii) based on easily ascertained information, and (iii) reported models’ final predictors. To investigate further the comparability of lifestyle predictors across models, estimates were standardised into risk ratios and visualised using forest plots.

**Results:**

From a total of 49 studies, 33 models were developed and 22 different existing models, mostly from Gail (22 studies) and Tyrer-Cuzick and co-workers (12 studies) were validated or modified for European populations. Family history of BC was the most frequently included predictor (31 models), while body mass index (BMI) and alcohol consumption (26 and 21 models, respectively) were the lifestyle predictors most often included, followed by smoking and physical activity (7 and 6 models respectively). Overall, for lifestyle predictors, their modest predictive contribution was greater for riskier lifestyle levels, though highly variable model estimates across different models.

**Conclusions:**

Given the increasing BC incidence rates in Europe, risk models utilising readily available risk factors could greatly aid in widening the population coverage of screening efforts, while the addition of lifestyle factors could help improving model performance and serve as intervention targets of prevention programmes.

**Supplementary Information:**

The online version contains supplementary material available at 10.1186/s12885-023-11174-w.

## Introduction

Breast cancer (BC) is the most frequently diagnosed cancer and the leading cause of cancer-related death among females in Europe, with nearly 580,000 new cases and 160,000 deaths in 2020; corresponding to one-third of the total cancer burden [1]. Incidence trends in Europe are mainly increasing due to multiple changes including hormonal and reproductive factors, increasing obesity and physical inactivity as well as increased screening intensity [2]. Population-based screening through mammography has contributed substantially to reductions in the mortality burden, as acknowledged by the evidence-based guidelines developed by the European Commission Initiative on Breast Cancer [3], as well as confirmed by a recent meta-analysis reporting reduction estimates ranging between 12 and 58% in screening attenders versus non-attenders [4]. At present, guidelines for early detection of BC, particularly those related to screening programmes, are targeting women between 45 and 74 years of age, hence seeing age as the main risk factor. Women identified to have a greater than average risk for BC due to a family history of BC or BC gene (BRCA) mutations, are normally subjected to personalised medical monitoring (outside of organised population-based screening programmes) [5]. However, besides accounting for these major non-modifiable risk factors, personalised risk-based screening accounting for individual modifiable risk factors, such as lifestyle, might be useful in detecting a greater number of early BC cases [6].

Numerous risk prediction models for BC, quantifying women’s future risk based on individual risk factors have been developed, as summarised in systematic reviews [7–11]. The most widely validated and utilised risk models that estimate future BC risk include the model of Gail and co-workers [12], developed in a US population, and the model of Tyrer-Cuzick (TC) and co-workers [13], developed in the UK and tailored to high-risk populations. Their original models focused mainly on age and non-modifiable hereditary (familial) variables, and hormonal and reproductive risk factors as predictors, because evidence of the BC risk modulation from modifiable lifestyle risk factors was not available at that time. Particularly, body fatness, alcohol consumption, smoking and physical inactivity, are now established BC risk factors by the Continuous Update Project (CUP), steered by the World Cancer Research Fund Network [14, 15]. Updated versions of these models as well as most newly developed models have, however, utilised modifiable lifestyle factors amongst other recently established risk factors, such as mammographic features [16, 17], and common genetic susceptibility variants [18–20] identified through Genome-Wide Association Studies (GWAS) and often aggregated in polygenic risk scores (PRS). The use of PRS for risk prediction intends to improve the understanding of genetic risks beyond the widely-used BRCA mutations, though with a debatable clinical utility [21].

Regardless of the model structure and predictors utilised, risk prediction models are undoubtedly relevant to facilitate risk stratification among the general population and provide the grounds for prevention strategies. While non-modifiable predictors, such as aging, and hereditary traits can be used to emphasise self-monitoring and close medical follow up, the modifiable risk factors, known to affect the onset of BC [14, 15], can be actionable targets for risk reduction in primary prevention strategies. In this regard, the main challenge relates to the applicability of individualised risk prediction models for BC in screening settings, beyond the clinical contexts where they have already been implemented [7]. This population-based approach calls for a simplification of risk prediction models emphasising the use of variables that are straightforwardly reported by the women or obtained from their medical files while acknowledging that laboratory- (e.g. genotyping) or imaging-based (e.g. mammograms) information are subject to data collection challenges, like time, cost-/health risk–benefit amongst others, and may not be available at the population level.

The aim of this review is to systematically assess population-based risk prediction models of primary BC based on easily obtained information, with a particular interest in understanding the contribution of established lifestyle-related risk factors [14, 15], that have been developed and/or validated for European populations, and including an evaluation of the risk of bias in model development and validation and predictive performances.

### Materials and methods

A systematic literature review was performed in accordance with the Preferred Reporting Items for Systematic Reviews and Meta-Analysis (PRISMA) guidelines during all stages of the design, implementation, and reporting of the systematic review [22], and registered in the PROSPERO database (CRD42021258286).

### Search strategy

An electronic literature search was performed in PubMed, Embase and Web of Science for the period between January 2000 to August 2022 using keywords and synonyms related to “breast cancer”, “risk”, and “model” and “prediction/assessment/estimation”. The search was complemented with hand searches of the citations of the retrieved systematic reviews and meta-analyses.

### Eligibility criteria

To be included in the systematic review, studies had to be published as a primary research paper in a peer-reviewed journal and either describe the development and/or the validation (performance assessment) of primary BC risk prediction model identifying groups or individuals at higher risk. Previous systematic reviews were only kept for reviewing cited papers. Model’s data source had to concern apparently healthy European females, from the general population, or females attending a preventive BC screening. The risk model had to utilise variables that are straightforwardly reported by the women or obtained from their medical files. Further, the following exclusion criteria were applied: prediction models accounting only for imaging- (e.g. mammographic features) and/or laboratory-based (e.g. PRS) information, as well as risk models not developed using classical regression, manuscripts reporting models developed for specific population subgroups (e.g. women with pre-existing (multi-) morbidity, with the exception of menopausal status); and conference proceedings, papers in languages other than English, and studies. Title and abstract screening, followed by a full-text review of the studies complying with the eligibility criteria were independently appraised by two investigators. Any discrepancy during the selection of the studies was resolved by consensus, and where necessary, group discussions among all investigators.

### Data extraction and synthesis

Data extraction for each risk prediction model was performed in duplicate using a standardised electronic excel template based on the framework of the CHARMS (critical appraisal and data extraction for systematic reviews of prediction modelling studies) checklist [23]. When the same study described multiple risk prediction models or applied multiple data sources for validation, each prediction model or data source was included separately.

Extracted information included: publication details (author, year, country, study name if available); study setting and population (source of data, country or region, sample size including total number and number of cases for development and/or validation, and if applicable by age group or menopausal status), outcome(s) to be predicted and timeframe of prediction; methods of model development (type of statistical model, variable selection method, missing data handling method); predicting variables (including the type and number of potential predictors considered and selected, and if available, reported regression coefficients and a measure of uncertainty, i.e. standard error (SE) or 95% confidence intervals (CI) for the selected lifestyle predictors); and, reported performance measures in internal or external validation for calibration (i.e., calibration plot, the ratio of observed to expected (O:E) probabilities, Hosmer–Lemeshow test), and discrimination (i.e., area under the receiver operating characteristic curve (AUROC)) where available.

Bias assessment was performed in parallel to data extraction, also in duplicate, and for both model development and validation, following the framework of PROBAST (Prediction model Risk Of Bias ASsessment Tool) [24], that allows the classification of each study as having a high, unclear or low risk of bias according to four domains: participants, predictors, outcome, and analyses. No studies were excluded based on risk of bias assessment alone.

### Model characteristics

Eligible studies and the characteristics of their prediction models developed and/or validated were qualitatively summarised in evidence tables. Visual comparisons were performed for included studies where lifestyle predictors coefficient estimates and their uncertainty were reported for model development studies, and for model validation studies, where model performance estimates and their uncertainty were reported.

### Visual comparison of lifestyle-related predictors

From the eligible studies, we identified lifestyle predictors employed in the different risk prediction models with established evidence as aetiological risk factors of BC, as reported from the CUP [14, 15]. After identifying those lifestyle factors with an explanatory and predictive character, retrieved coefficient estimates and their uncertainty were standardised to be visually compared in forest plots, stratified according to their choice of comparison; for continuous variables per x-level increment, and for categorical variables the contrast between groups, using the middle and the extremes versus the lowest risk state if more than two groups were available. The type of the regression-based estimates varied between the studies included in our systematic review, hence the conversion of odds ratios (ORs) and hazard ratios (HRs) into a risk ratio (also known as relative risk) (RR) was necessary for visual comparability. All non-RR point estimates were converted to RR using one of the following equations:

$$RR= \frac{OR}{\left(1-{p}_{0}\right)+({p}_{0}*OR)}$$

or

$$RR= \frac{1-{e}^{HR*\mathrm{ln}(1-r)}}{r}$$

where *p*_*0,*_ and *r* represents the baseline risk and the incidence rate, respectively, of the outcome for the reference group or in the absence of information for the reference group, the incidence proportion or rate for the overall study population. Similarly, the retrieved estimates of model calibration (i.e., O:E ratios) and discrimination (i.e., c-statistics) were visualised in forest plots for comparatively review of the performance of the risk prediction models included. Forest plots were plotted in R version 4.1.2 using the package *meta* [25].

## Results

The initial search yielded 25,499 articles, and after removing duplicates 14,959 articles were screened yielding 427 articles to be retrieved for full-text review. After the exclusion of 371 articles due to varied reasons (Supplementary Fig. 1), and an additional inclusion of 7 full-text articles identified through hand searching from citations (i.e. from 37 previously published literature reviews and meta-analyses), a total of 49 studies were included in the present review. A detailed description of these eligible studies is provided in Table 1 and Supplementary Table 1, and includes 21 studies describing 33 risk prediction models developed for a European population, and 28 studies reporting the 105 validation and/or modification of a model developed elsewhere in a European population. Altogether, a total of 130 existing models (from 35 studies) were validated and/or modified, with the Gail (22) and TC (12) models as the most frequently used. Most studies were conducted using data from Western or Southern (18 and 13 studies, respectively) Europe, with the studies from Southern Europe more often applied for developing a prediction model, and those from Western Europe for validating/modifying an existing model. When assessing risk of bias according to PROBAST, most studies were considered to carry a high risk of bias for the domain of analyses (21 studies), due mainly to an a priori defined set of predictors (39), inadequate handling of missing data (36), incomplete report of the relevant performance measures (i.e., only calibration or only discrimination instead of both, or none; 27), lack of accounting for model overfitting and optimism (25), no report of the final model (10) and insufficient sample size (6).

**Table 1:** Summary of eligible studies describing risk prediction models for the incidence of primary breast cancer, applicable to European populations^a^

		Studies developing new EUR prediction model			Studies validating existing model	
	All studies	As single purpose	+ validating existing model	+ modifying existing model	As single purpose	+ modifying existing model
General study characteristics						
N studies	49	14	3	4	5	23
N models developed	33	NA	NA	NA	NA	NA
N models validated	52	NA	NA	NA	NA	NA
N models modified	78	NA	NA	NA	NA	NA
N studies applying existing						
Gail model	22	NA	3	4	3	12
TC model	12	NA	1	2	1	8
Geographic location						
Northern EUR	5	0	1	2	0	2
Central and Eastern EUR	6	1	0	1	0	4
Southern EUR	13	6	0	1	2	4
Western EUR	18	4	1	0	3	10
Across continent(s)	7	3	1	0	0	3
Risk of bias (PROBAST)						
Domain 1: Participants						
Risk of bias, high	0	0	0	0	0	0
Data sources						
Prospective cohort	12	2	3	1	2	4
Case-cohort or nested case–control studies	16	4	1	2	0	9
Case–control studies	15	6	0	2	0	7
RCTs	1	0	1	0	0	0
Surveys or registries	10	3	0	1	3	3
Domain 2: Predictors						
Risk of bias, high	0	0	0	0	0	0
Predictor unavailability at model use	9	2	0	0	0	7
Domain 3: Outcome						
Risk of bias, high	1	0	0	0	0	1
Domain 4: Analysis						
Risk of bias, high	21	11	1	3	2	4
Sample size, cases > 100	6	0	1	1	3	1
Missing data						
Complete Cases	29	8	1	1	4	15
NS	7	3	0	3	0	1
Variable selection						
A priori defined	39	8	1	4	5	21
Uni- before multivariate	2	2	0	0	NA	NA
Incomplete model performance estimates	27	10	1	3	2	11
No adjustment for overfitting and optimism	25	10	1	2	NA	12
No report of final model	10	6	1	0	NA	3

^a^Studies were categorised into five types: studies developing a new prediction model in a European population (either having this as single purpose of the study or next to this also validating or modifying an existing model) and studies validating an existing model (either having this as single purpose of the study or next to this also modifying an existing model)

Abbreviations: *EUR* Europe with Northern EUR including studies from Sweden, Central and Eastern from Czech Republic, Germany and Poland, Southern from Cyprus, Italy, Spain and Turkey, Western from the UK, France and the Netherlands, and across continent(s) including studies from Europe only as well as from Europe, Australia and/or the United States, *NA* not applicable, *NS* not specified, *PROBAST* Prediction model Risk Of Bias Assessment Tool, including evaluation on the domain of Participants (P), Predictors (P), Outcome (O) and Statistical Analyses (A) *RCT* Randomised controlled trial, *TC* Tyrer-Cuzick risk model (or IBIS risk tool)

### Variables included in the risk prediction models

Predictors utilised in the eligible BC risk prediction models are shown in Table 2 and Supplementary Table 2, presenting the models developed in a European population, and Supplementary Table 3, presenting models validated in and/or modified for a European population. The number of predictors in the initial models varied from 3 [26] up to 12 [27, 28], and even more in the extended BOADICEA model of Lee and co-workers [29].

**Table 2:** Predictors included in the final models of the 33 breast cancer risk prediction models developed to European populations

Author, year	Country	Model name, if available	DG	Medical history				Lifestyle					
				Fam	Personal								
					Gen	ReHo	(B) dz	Anthro		LS risk behaviours			
								BMI	H	A	PA	SM	D
Tyrer, et al., 2004 [13]	GBR	-	X	X	X	X	X	X	X				
Boyle et al., 2004 [30]	ITA	M-Nutrient, < 50y (M1)		X		X				X			X
		M-Nutrient, ≥ 50y (M2)		X		X		X		X	X		X
		M-Food, < 50y (M3)		X		X				X			X
		M-Food, ≥ 50y (M4)		X		X		X		X	X		X
Petracci, et al., 2011 [31]	ITA	5NMRF + 2MRF M (M1)	X	X		X	X	X		X	X		
Hüsing et al., 2012 [32]	EUR, USA	Covariate M (M1)				X		X		X		X	
Rauh et al., 2012 [33]	DEU	RFs M (M1)	X			X		X					
Dartois, et al., 2015 [34]	FR	kNN-PrM (M1)	X	X		X	X						
		kNN-PoM (M2)	X	X		X	X						
		Cox-PrM (M3)	X	X			X						
		Cox-PoM (M4)	X	X		X	X	X		X			
Hippisley-Cox, et al., 2015 [35]	GBR	QCancer (M1)	X	X		X	X			X			
Maas et al., 2016 [36]	EUR, AUS, USA	NM-MRFs (iCARE) M1)		X		X		X	X	X		X	
Eriksson, et al., 2017 [37]	SWE	MammoDetect-PrM (M1)		X		X	X	X					
		MammoDetect-PoM (M2)		X		X	X	X					
Dierssen-Sotos et al., 2018 [38]	ESP	MRFs (M1)				X		X		X			
		NMRFs (M2)	X	X		X			X				
Gabrielson et al., 2018 [39]	SWE	PrM-M (M1)		X		X	X						
		PoM-M (M2)		X		X	X	X				X	
Li et al., 2018 [27]	EPIC	ER + M (M1)				X		X	X	X			
		ER- M (M2)				X		X	X	X			
		Omnibus M (M3)				X		X	X	X			
Lumachi et al., 2018 [40]	ESP		X			X	X						
Rudolph et al., 2018 [41]	EUR, AUS, USA	NM-MRFS (M1)				X		X	X	X			
Lee et al., 2019 [29]	GBR	Extended BOADICEA (M1)	X	X	X	X	X	X	X	X			
Usher-Smith et al., 2019 [26]	GBR							X		X	X		
Eriksson, et al., 2020 [42]	SWE	KARMA + RFs-PrM (M1)		X			X	X		X		X	
		KARMA + RFs-PoM (M2)		X		X	X	X		X		X	
Triviño et al., 2020 [43]	ESP	-	X	X	X	X	X						
Bonnet et al., 2021 [44]	FRA	-	X	X		X	X						
Louro et al., 2021 [45]	ESP	-	X	X			X						
Yiangou et al., 2021 [28]	CYP	-	X	X	X	X		X	X			X	

Abbreviations: *Anthro* Anthropometrics, including body mass index (BMI) and height (H); AUS, Australia; (B) dz, pre-existing (breast) diseases, *BMI* Body mass index, *BOADICEA* Breast and Ovarian Analysis of Disease Incidence and Carrier Estimation Algorithm, *CYP* Cyprus, *DEU* Germany, *DG* Demographics, *EPIC* European Prospective Investigation into Cancer and Nutrition Study with cohorts from Denmark, France, Germany, Greece, Italy, Norway, Spain, Sweden, the Netherlands, United Kingdom; ER + /ER-, oestrogen receptor-positive/-negative breast cancer, *ESP* Spain, *EUR* Europe, *GBR* Great Britain (UK), *Gen* Genetic information, *Fam* Family history of (breast) cancer, *ITA* Italy, *kNN* k-nearest neighbourhood algorithm, *LS* Lifestyle, *LS* Risk behaviours, including alcohol consumption (A), physical activity (PA), smoking (SM) and diet (D), *M* Model, *MRFs* Modifiable risk factors, *NMRFs* Non-modifiable risk factors, *PoM* Post-menopausal women, *PrM* Pre-menopausal women, *ReHo* Reproductive and hormonal risk factors, *SWE* Sweden, *USA* United States of America

Generally, predictors were categorised into seven types: demographics, medical history (family history of cancer and personal medical history, including genetics, reproductive and hormonal factors, and pre-existing (breast) diseases and related parameters), and lifestyle (anthropometrics, and lifestyle risk behaviours, including alcohol, diet, physical activity and smoking). From the list of variables selected in the risk prediction models, the most commonly identified predictor was family history of BC, mostly operationalised as (the number of) first-degree relatives, for both the European developed models (24 models; Table 2) and the models validated in/modified for a European population (18 of which 9 were from non-European origin; Supplementary Table 3).

Of the European-developed models (as presented in Table 2), other most commonly identified predictors were menopausal hormone therapy (21 models), body mass index (BMI; 21), age at menarche (19), alcohol consumption (18), age at first living birth (16), age at time of study (14), percent mammographic density (PMD; 11), parity (11) and history of benign breast disease (10). Of the models of non-European origin that are validated in/modified for a European population (as presented in Supplementary Table 3), other most commonly identified predictors were age at time of study (8), history of benign breast disease (7), age at first living birth (6), followed by age at menarche, PMD and BMI (5 each), and parity and menopausal hormone therapy (4 each). More recently developed models as well as modified versions of existing risk prediction models incorporated more often PMD (15 original and 5 modified) and/or PRS (3 and 9, respectively) as predicting variables. Apart from BMI (as well as alcohol consumption for the European-originated models), modifiable lifestyle-related risk factors were considered as predictors only in a limited number of models, with the most shared being smoking (included in 6 European and 1 non-European) and physical activity (in 4 and 2, respectively, and most often represented as leisure-time physical activity). Diet-related factors were selected as predictors in only five models and were operationalised by the number of daily portions of fruit and vegetables (in 3 models) and by a composite risk score from intake of beta-carotene and vitamin E (in 2).

### RR estimates of modifiable lifestyle risk predictors for BC

For a visual comparison of model-specific estimates of the modifiable lifestyle predictors recognised by the CUP programme as aetiological factors with probable or convincing evidence (i.e., BMI, alcohol consumption and physical activity), data from 24 studies representing 32 different risk prediction models, irrespective of their origin, were eligible for visual comparison (Supplementary Table 4). Excluded were 5 models from 2 studies [33, 34] because of unavailable regression coefficients, whereas 6 models from 6 studies had predictors with unavailable measure of uncertainty [29, 39, 46–48].

**Fig. 1:**
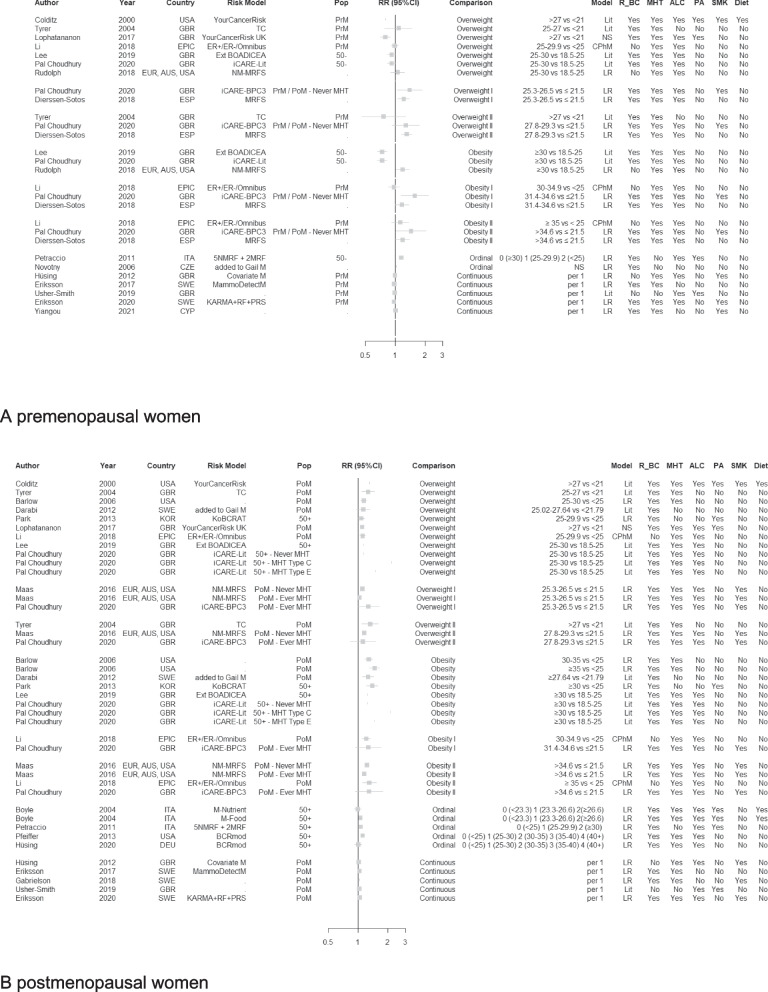
Forest plot of standardised estimates (RR and corresponding 95% confidence intervals) of body mass index as predictor across risk prediction models, stratified by premenopausal status by premenopausal status (A premenopausal women and B postmenopausal) and their choice of comparison group Footnote 1: For each available RR estimate with its choice of comparison group, the following information was included: publication details (author, year and country, and if applicable model name and population (Pop) suitable for the model), type of statistical model (either literature-based (Lit), Cox (CPhM) or logistic (LR) regression models), and the inclusion of pre-selected predicting variables (i.e., relatives of breast cancer (R_BC), menopausal hormone therapy (MHT), body mass index (BMI), alcohol consumption (ALC), physical activity (PA), smoking (SMK), and any diet-related predictors (Diet). Footnote 2: Studies not reporting 95% confidence intervals nor standard errors were the following: Colditz et al., 2000; Lee et al., 2019; Pal Choudhury et al., 2020; Novotny et al., 2006 (see also Supplementary Table 4) Abbreviations: *AUS* Australia, *BOADICEA* Breast and Ovarian Analysis of Disease Incidence and Carrier Estimation Algorithm, with Ext for Extended version, BCRmod, (Ko)BCRAT, Breast Cancer Risk Assessment Tool (Gail), *BMI* Body mass index in kg/m2, with ‘Overweight’ defined as BMI above 25 and ‘Obesity’ above 30, and additional ‘Overweight I and II’ and ‘Obesity I and II’ specifying further subdivision, *BPC3* Breast and Prostate Cancer Cohort Consortium, *CYP* Cyprus, *CZE* Czech Republic, *DEU* Germany, *ESP* Spain, *EPIC* European Prospective Investigation into Cancer and Nutrition study, *EUR* Europe, ER ± , oestrogen receptor-positive/-negative breast cancer, *GBR* Great Britain (UK), *ITA* Italy, *KARMA* Karolinska Mammography Project for Risk Prediction of Breast Cancer cohort, *KOR* South Korea, *M* Model, *NM-MRFs* Non-modifiable and modifiable risk factors, *POL* Poland, *PoM* Post-Menopausal women, *PrM* Pre-Menopausal women, *PRS* Polygenic risk score, *RF* Risk factor, *RR* Relative risk, *SWE* Sweden, *TC* Tyrer-Cuzick risk model (or IBIS risk tool), *USA* United States of America

The model-specific RR estimates for BMI in categories showed a noticeable variation (Fig. 1), particularly in premenopausal women ranging from 0.77 (95%CI: 0.47–1.28) to 1.39 (1.04–1.85) for overweight, and from 0.97 (0.85–1.10) to 1.44 (1.02–2.05) for obesity, and in postmenopausal women from 1.03 (1.02–1.04) to 1.32 (1.07–1.57) for overweight, and from 1.15 (1.13–1.18) to 1.47 (1.30–1.64) for obesity. The predictive RR estimates of BMI as a continuous variable were found to be of the same magnitude, notwithstanding the limited number of risk model equations using continuous RR for BMI. Taking the middle value of RR estimates and its corresponding 95%CI, median continuous RR per unit increment in BMI was 0.99 (0.98–1.01) for premenopausal and 1.03 (1.01–1.05) for postmenopausal women.

**Fig. 2:**
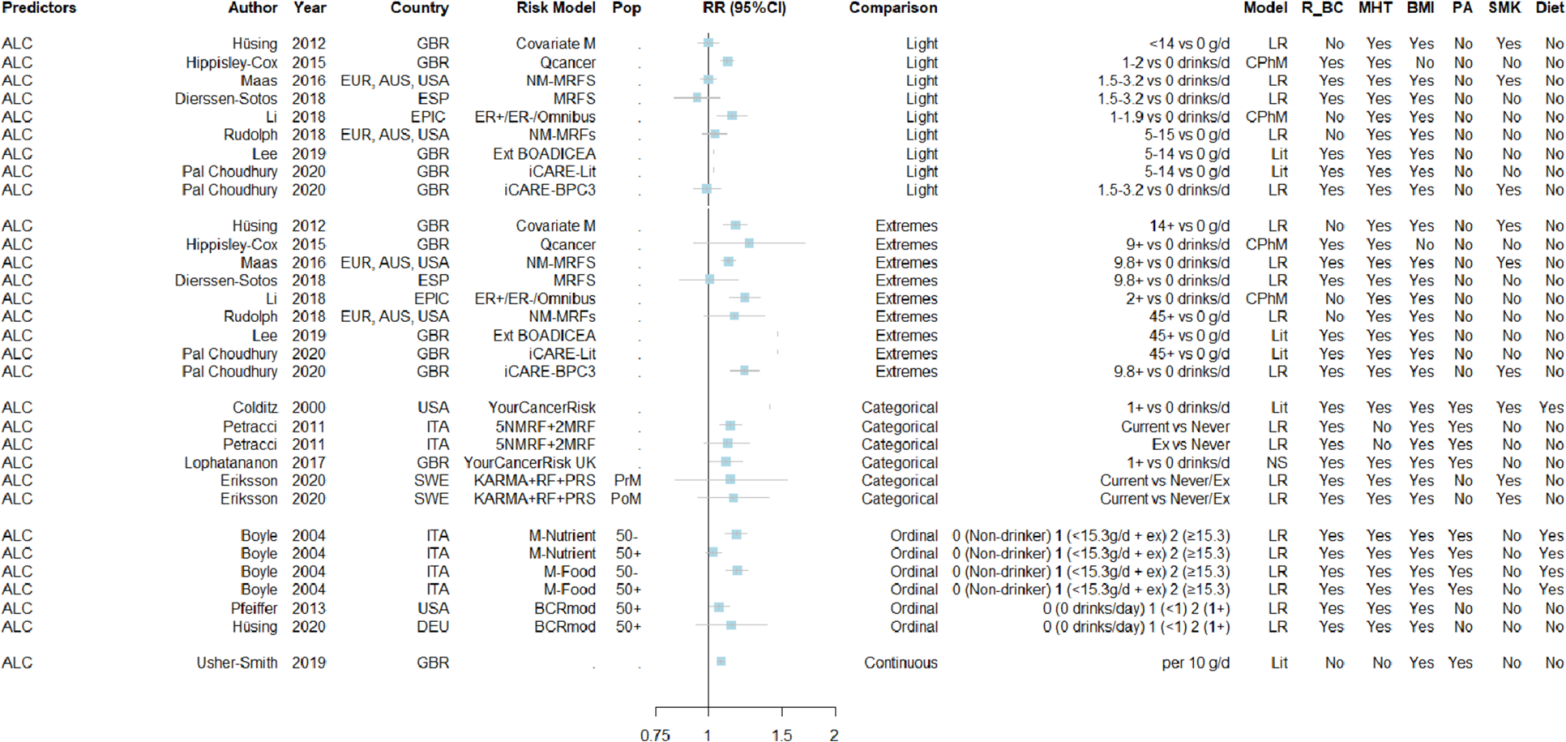
Forest plot of standardised estimates (RR and corresponding 95% confidence intervals) of alcohol consumption as predictor across risk prediction models, stratified by their choice of comparison group Footnote 1: For each available RR estimate with its choice of comparison group, the following information was included: publication details (author, year and country, and if applicable model name and population (Pop) suitable for the model), type of statistical model (either literature-based (Lit), Cox (CPhM) or logistic (LR) regression models), and the inclusion of pre-selected predicting variables (i.e., relatives of breast cancer (R_BC), menopausal hormone therapy (MHT), body mass index (BMI), alcohol consumption (ALC), physical activity (PA), smoking (SMK), and any diet-related predictors (Diet). Footnote 2: Studies not reporting 95% confidence intervals nor standard errors were the following: Colditz et al., 2000; Pal Choudhury et al., 2020 (see also Supplementary Table 4) Abbreviations: *AUS* Australia, *BOADICEA* Breast and Ovarian Analysis of Disease Incidence and Carrier Estimation Algorithm, with Ext for Extended version, *BCRmod* Breast Cancer Risk Assessment Tool (Gail), *BPC3* Breast and Prostate Cancer Cohort Consortium, *DEU* Germany, *ESP* Spain, *EPIC* European Prospective Investigation into Cancer and Nutrition study, *EUR* Europe, *ER* ± Oestrogen receptor-positive/-negative breast cancer, *GBR* Great Britain (UK), *ITA* Italy, *KARMA* Karolinska Mammography Project for Risk Prediction of Breast Cancer cohort, *M* Model, *NM-MRFs* Non-modifiable and modifiable risk factors, *PoM* Post-Menopausal women, *PrM* Pre-Menopausal women, *PRS* Polygenic risk score, *RF* Risk factor, *RR* Relative risk, *SWE* Sweden, *USA* United States of America

**Fig. 3:**
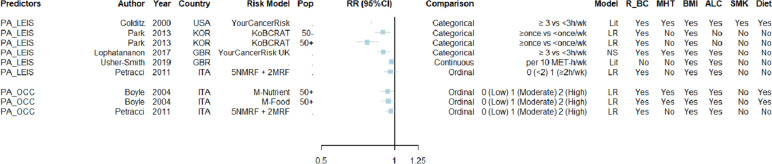
Forest plot of standardised estimates (RR and corresponding 95% confidence intervals) of physical activity as predictor across risk prediction models, stratified by their choice of comparison group Footnote 1: For each available RR estimate with its choice of comparison group, the following information was included: publication details (author, year and country, and if applicable model name and population (Pop) suitable for the model), type of statistical model (either literature-based (Lit), Cox (CPhM) or logistic (LR) regression models), and the inclusion of pre-selected predicting variables (i.e., relatives of breast cancer (R_BC), menopausal hormone therapy (MHT), body mass index (BMI), alcohol consumption (ALC), physical activity (PA), smoking (SMK), and any diet-related predictors (Diet). Footnote 2: Studies not reporting 95% confidence intervals nor standard errors were the following: Colditz et al., 2000 (see also Supplementary Table 4) Abbreviations: *GBR* Great Britain (UK), *ITA* Italy, *KoBCRAT* Breast Cancer Risk Assessment Tool (Gail), *KOR* South Korea, *M* Model, *NM-MRFs* Non-modifiable and modifiable risk factors, *RR* Relative risk, *USA* United States of America

**Fig. 4:**
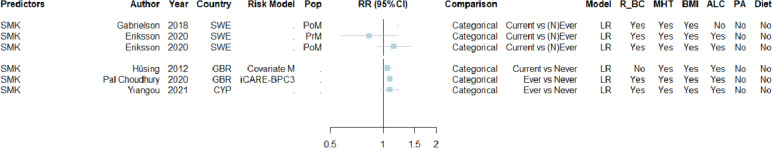
Forest plot of standardised estimates (RR and corresponding 95% confidence intervals) of smoking status as predictor across risk prediction models, stratified by their choice of comparison group Footnote 1: For each available RR estimate with its choice of comparison group, the following information was included: publication details (author, year and country, and if applicable model name and population (Pop) suitable for the model), type of statistical model (either literature-based (Lit), Cox (CPhM) or logistic (LR) regression models), and the inclusion of pre-selected predicting variables (i.e., relatives of breast cancer (R_BC), menopausal hormone therapy (MHT), body mass index (BMI), alcohol consumption (ALC), physical activity (PA), smoking (SMK), and any diet-related predictors (Diet) Footnote 2: Studies not reporting 95% confidence intervals nor standard errors were the following: Gabrielson et al., 2018 (see also Supplementary Table 4) Abbreviations: *BPC3* Breast and Prostate Cancer Cohort Consortium, *CYP* Cyprus, *GBR* Great Britain (UK), *M* model, *NM-MRFs* Non-modifiable and modifiable risk factors, *PoM* Post-Menopausal women, *PrM* Pre-Menopausal women, *SWE* Sweden, *RR* Relative risk

The predictive contribution of alcohol consumption (Fig. 2), leisure-time physical activity (Fig. 3) as well as smoking (Fig. 4) showed noticeable variation across risk prediction equations. Particularly, for alcohol, model-specific RRs ranged from 0.99 (0.92–1.07) to 1.14 (1.05–1.24) for light drinkers, from 1.01 (0.84–1.16) to 1.25 (0.92–1.70) for heavy drinkers, and from 1.03 (0.98–1.07) to 1.17 (1.10–1.24) when treating the ordinal scale as continuous. For physical activity, it varied from 0.77 (0.69–0.85) to 0.97 (0.95–0.98), and for smoking from 0.83 (0.58–1.20) to 1.14 (0.91–1.41) when comparing current versus (n)ever smokers. However, the model-specific RR estimates were of similar magnitude for occupational physical activity (median of 0.94 (0.88–1.00) per unit increment on a 0 to 2 ordinal scale) and for smoking when comparing ever versus never (1.09 (0.96–1.22)).

### Modifications to existing BC risk prediction models

A total of 35 studies were identified describing the external validation and/or modifications of an existing risk prediction model for BC in the European population, either considering solely external validation (8 studies) or modification(s) as well (27). Supplementary Table 3 provides an overview of the 35 studies describing an external validation of and/or a modification to existing BC risk prediction models, applicable to European populations. The modifications made to the existing risk prediction models were: the inclusion of additional (predicting) variables (27), the update of coefficients (i.e., relative risks; 17 of which 10 updated original predicting variables and 11 (also) updated the additional included variables), and the adjustment of baseline risk/hazard (i.e., underlying BC incidence rates and competing mortality rates; 9).

Predictors most often added to existing risk models were PRS (18 studies), followed by PMD (8) and hormonal biomarkers (4), while additional modifiable lifestyle factors of BMI and alcohol consumption were added to original models of Gail [49] and BOADICEA [29]. Of the identified validation studies in European populations, the Gail model (and its updates) was the existing BC risk prediction model that was considered the most (22), with the majority of the studies also modifying the model (11 including additional variables, 11 updating the original coefficients, and 8 adjusting baseline risk/hazard). Furthermore, the TC model, also called the IBIS risk tool, was considered in a total of 12 studies, all conducted in a UK or Sweden-based cohort aligning with its assessment calculator that has available underlying competing mortality with UK and Sweden rates.

### Estimates of model performances

Supplementary Figs. 2–5 show the visual presentation of the model performance of the risk prediction models. A total of 20 studies (accounting for 69 estimates of O:E ratio and/or c-statistic) measured the performance of the Gail model in a European population (Supplementary Fig. 2). Good calibration (defined as an O:E ratio between 0.8 and 1.2) was seen for 18 (81.8%) out of the 22 available estimates for O:E ratio. None of the available estimates were observed to have good discrimination (defined as a c-statistics above 0.75), as a c-statistic between 0.5 and 0.6 was reported for the majority of them (i.e., 45 (81.8%) out of 55 available estimates for c-statistics). Of the 6 other existing non-European developed risk prediction models that were validated in a European population, only estimates of c-statistics were available for both the development study as the European validation study, showing poorer discrimination in the latter (Supplementary Fig. 5). The IBIS risk tool was applied in 11 studies (accounting for 36 estimates of O:E ratio and/or c-statistic; Supplementary Fig. 3) with 11 (76.9%) out of the 13 available estimates for O:E ratio suggesting good calibration, and one estimate suggesting good discrimination, instead a c-statistics between 0.6 and 0.75 was reported for the majority of them (i.e., 18 (69.2%) out of the 26 available estimates). Supplementary Fig. 4 shows the model performance of the other 22 European risk prediction models identified, with 16 studies (accounting for 30 models, including modified versions) available for internal validation and 8 studies (accounting for 14 models, including modified versions) for external validation. Similarly, estimates of the O:E ratio pointed to good calibration for most of them (i.e., 21 (80.8%) out of the 26 available estimates), but were also less available than those of the c-statistic. Available estimates of the c-statistics pointed to good discrimination for only 2 of them, with the majority being between 0.6 and 0.75 (i.e., 67 (75.2%) out of the 89 estimates available), and 20 estimates (22.5%) between 0.5 and 0.6. Where made, updates to the existing risk prediction model did not appear to improve model performance estimates much.

## Discussion

This systematic review summarised the evidence published over the last two decades on primary BC risk prediction models with straightforwardly ascertained predictors, including lifestyle factors, applicable to European populations. From the list of predictors reviewed, family history was the most frequently included, while, apart from other non-modifiable predictors (such as genetic predisposition, reproductive and hormonal factors), the commonly shared modifiable lifestyle-related risk factors were BMI and alcohol consumption, followed by smoking and physical activity, and more scarcely diet-related variables. Evaluating the validation studies included, all risk models leaned towards strong calibration, but low discriminatory accuracy, implying a good performance for predicting risk at a population level, but not at the individual level.

The European Breast Guidelines, coordinated by the European Commission’s Joint Research Centre, provide evidence-based recommendations for BC screenings. These guidelines suggest that mammography screening should be initiated based on age and the presence of specific risk factors, including genetic predisposition (mutations in BRCA1/2), reproductive history (such as age at first birth, reproductive interval index, and parity), and race/ethnicity [3, 50]. These risk factors have been identified through sound scientific evidence and are important considerations for determining the appropriate time to start mammography screenings. However, these guidelines do not provide any recommendation for the use of currently available risk prediction models for risk stratification, although a number of them have already been developed and validated for European populations, with most of them utilising solely predictors that are easily ascertained through patients’ interviews, as identified through this literature review. Further, risk-stratified breast screening using multifactorial risk assessment is increasingly regarded as a promising approach for targeted intensification of preventive measures and of early detection, in particular for the identification of high-risk individuals who would benefit the most from participating in preventive and/or screening programmes [6, 51, 52]. However, the actual implementation of such an individualised risk estimation approach needs further investigations into its feasibility and acceptability by the healthcare system and the target population amongst other relevant stakeholders [53, 54].

Prediction of BC might be challenged by its multifactorial occurrence where genetic susceptibility interacts with non-genetic (hormonal/reproductive history, environmental, lifestyle) factors [55], and hence best practice is to include many of them in the variable selection to obtain a model explaining the greatest amount of variance [56]. Including (previously identified) causal factors as predictors may enhance the credibility and uptake of the model across various settings and populations [57], and particularly those modifiable ones could contribute to improved prevention through motivating lifestyle change [55]. At present, some of the existing widely-known BC risk prediction models, like Gail [49] and BOADICEA [29], have been updated to include lifestyle factors, while also most of the risk prediction models based on classical factors (like demographics, and family and personal medical history) are inclined to utilise at least one lifestyle factor, as noted by our literature review. Of the lifestyle factors, the most frequently included in risk prediction were BMI and alcohol consumption, and to a lesser extent smoking and physical activity, with all of them also being identified as convincing/probable causal risk factors of BC by the CUP [14]. Interestingly, only five models introduced dietary factors as predictors of BC, aligned with current guidance [14], despite the growing body of evidence pointing to a determining role of healthier diets in the prevention of BC [58], suggesting a need of further research in this area. Whether the inclusion of lifestyle factors resulted in improved discriminatory accuracy as compared to (previous) risk prediction models solely based on classical irreversible risk factors remains questionable. Reported c-statistics were close to 0.6 for almost all models, with barely any improvements for the modifications made, while O:E ratios were close to 1.0 for most models, consistent with previous reviews [7–11]. However, it should be noted that the assessment of lifestyle factors is susceptible to response bias, i.e., predominantly social desirability bias, potentially limiting the impact that the addition of lifestyle factors has on overall model accuracy. Even though, the aim of a risk prediction model is to accurately identify high-risk individuals based on multiple factors, irrespective of their causal association with the outcome, the inclusion of established aetiological modifiable risk factors could serve a double purpose: improving the predicting accuracy of the model and constituting an actionable target for preventive strategies [56, 57].

Generally, risk models for BC, as identified by our and previous reviews [7–11], are inclined to be more suitable for predicting the BC incidence within a population rather than an individual’s risk. This may be explained by the likely oversimplification of complex relationships and of non-linear interactions in numerous risk factors in risk models applying the classical (logistic or Cox) regression. In light of this, ML techniques have been attracting a lot of interest for their potential use in prediction [59], including individualised BC risk prediction [60]. ML-based BC risk prediction models were shown to have better discriminatory accuracy, albeit substantial heterogeneity, as evaluated in a head-to-head comparison with the classical models [60]. They are, however, often referred to as black box models with known inputs and outputs but unidentified in-between processes, and this not only hinders model reliability and clinical feasibility [60], but might also lead to faulty decision-making [61]. In future investigations, models should be inherently interpretable, and preferably built by following established guidelines for development, validation and update [62–64] as well as for reporting [65, 66], aimed to deliver models that achieve high discrimination and are well-calibrated as evaluated by unbiased estimates for predictive performance.

Notably, the analytical domain of developing and validating risk prediction models could be improved since most studies in our review were considered to carry a high risk of bias. This high risk of bias was mainly due to the selection of predictors, handling of missing data, corrections for optimism and overfitting, and incomplete performance measures. In addition, the reporting of the development and validation of the prediction models showed room for improvement, and the various means of reporting practices often hindered proper bias assessment. Currently, there is a standard for reporting available, i.e., the Transparent Reporting of a multivariable prediction model for Individual Prognosis Or Diagnosis (TRIPOD) [65, 66], and following this set of reporting guidelines should improve the quality of reporting of studies developing/validating/updating predictive models. Yet prediction model studies arising after the publication of the TRIPOD statement remain poorly conducted and poorly reported [67], in the present literature review, just one study mentioned to have followed the guidelines during model development and validation [26]. With the increasing need for efficient use of risk prediction models in clinical decision making, there urgently needs to be greater research efforts into optimising the ease of use of and adherence to the reporting guidelines.

Evidence synthesis of risk prediction models plays a key role in interpretating their potential applicability and generalisability across different settings and populations. In this regard, compared to the established non-modifiable BC-related risk factors, such as family history, genetic, reproductive and hormonal risk factors, and pre-existing breast-related diseases, lifestyle risk factors might provide an avenue for BC risk models that may motivate lifestyle change at an individual level, even though at present the added value of integrating them remain unanswered. Likewise, in this study, the retrieved estimates of RR for lifestyle risk factors could not be summarised into a weighted “meta-analysed” average, because of substantial heterogeneity across risk models concerning the predicting variables included as well as underlying model assumptions. Further studies are therefore warranted to evaluate whether employing lifestyle risk factors beyond the classical risk factors are valuable for the identification of individuals at risk for incident BC, and subsequently contribute to improved prevention through participation in screening and lifestyle programmes at the individual level.

In conclusion, BC is a prevalent disease, and while screening programs exist, they are not infallible. Developing a risk prediction model that estimates an individual's risk of developing BC using readily available risk factors could greatly aid in widen the population coverage of the programmes, while the inclusion of lifestyle factors could help improving model’s performance and serve as intervention targets. Further, an enhanced effective BC risk prediction model should prioritise ensuring methodological quality by using data sources with sufficient sample sizes, applying multiple imputation for missing data, using appropriate variable selection approaches, adjusting for model fitting and optimism, and measuring both calibration and discrimination. This screening approach would help shifting the population towards less prevalent lifestyle risk factors while improving the accuracy and clinical relevance of the model.

## Supplementary Information

**Additional file 1. **

**Additional file 2. **

## Data Availability

All data, including the references of the published data, generated or analysed during this study are included in this published article and its supplementary information files.
