# Supplementary material for: Understanding the contribution of lifestyle in breast cancer risk prediction: a systematic review of models applicable to Europe
Source: BMC Cancer. 2023 Jul 21;23:687. doi: 10.1186/s12885-023-11174-w (PMC10360320; doi:10.1186/s12885-023-11174-w)
Supplement: Supplementary file 1 — Additional file 1. [file 12885_2023_11174_MOESM1_ESM.docx]

Supplementary Figures

**Identification of studies via databases and registers**

Records identified through database searching:

PubMed (n =10,681)

Web of Science (n = 3,895)
Embase (n=10,923)

Records removed:

Duplicate records (n =10,540)

**Identification**

Records screened

(n =14,959)

Records excluded
based on title or abstract review

(n =14,532)

Reports sought for retrieval

(n =427)

Reports not retrieved

(n = 68)

**Screening**

Reports excluded with reasons:

Publication type (n = 51)
Study aim (n = 112)

Population (n = 49)

Predictors (n = 65)

Outcome (n = 15)
Analyses (n = 11)

Reports assessed for eligibility

(n = 359)

Reports additionally identified through hand searches from reference list of reviews and meta-analyses (n = 37): n = 7

Studies included in the review

(n = 49)

**Included**

**Supplementary Figure 1** Flow chart of studies included in the review

*From:*  Page MJ, McKenzie JE, Bossuyt PM, Boutron I, Hoffmann TC, Mulrow CD, et al. The PRISMA 2020 statement: an updated guideline for reporting systematic reviews. BMJ 2021;372:n71. doi: 10.1136/bmj.n71

For more information, visit: <http://www.prisma-statement.org/>


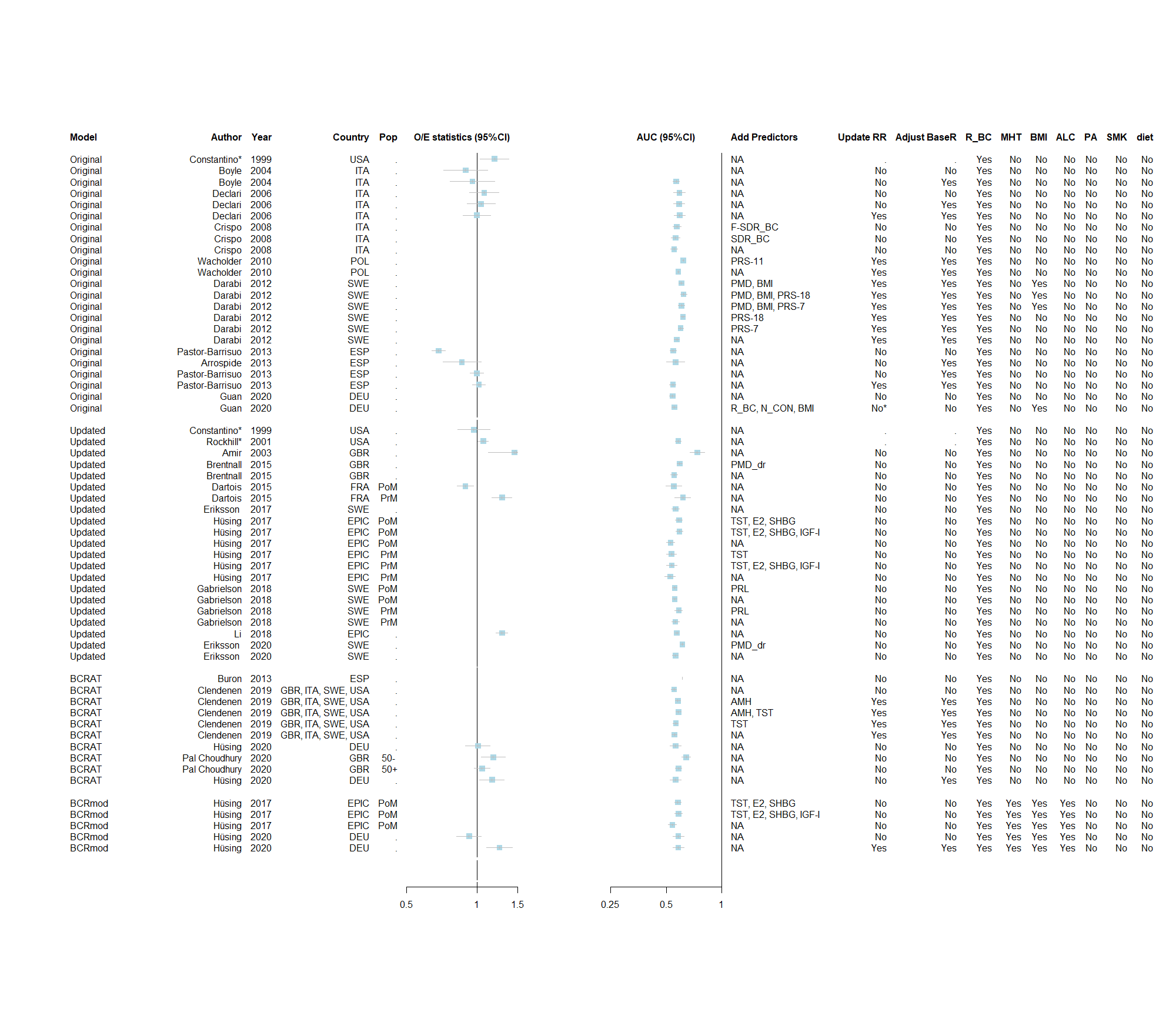


**Supplementary Figure 2** Predictive performance (as measured by the O:E ratio and/or c-statistic) of the Gail breast cancer risk prediction models validated in European populations.


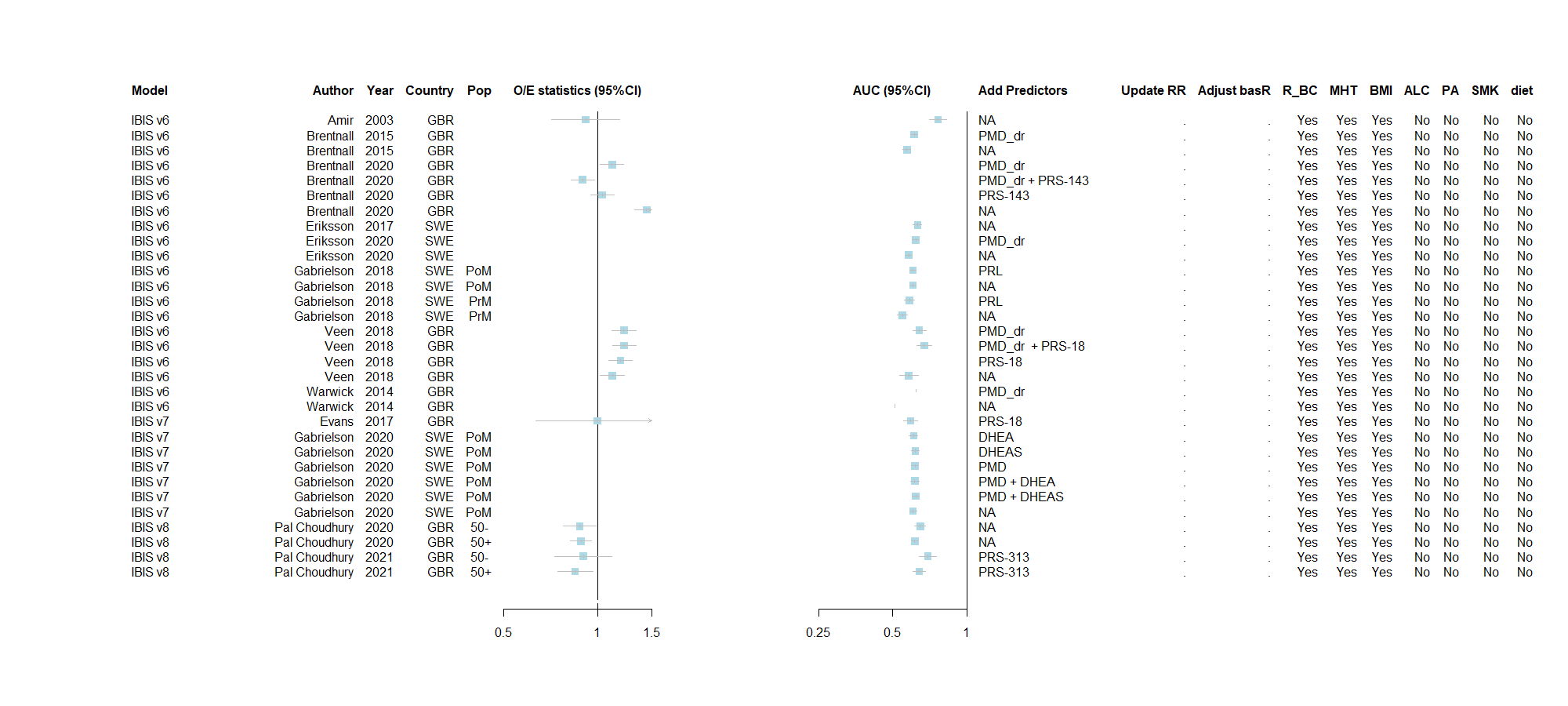


**Supplementary Figure 3** Predictive performance of (as measured by the O:E ratio and/or c-statistic) of the Tyrer-Cuzick Breast cancer risk prediction model validated in European populations.


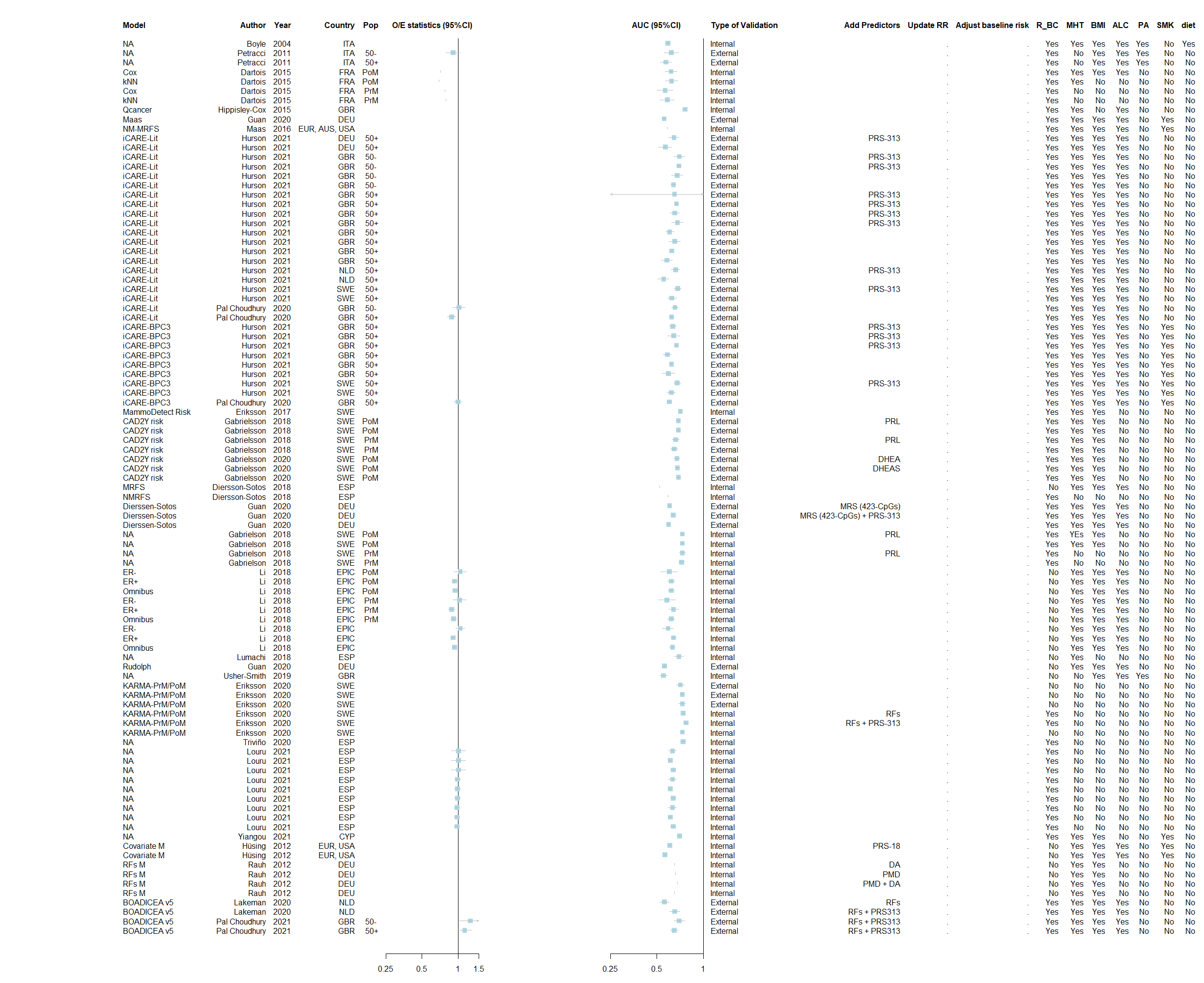


**Supplementary Figure 4** Predictive performance (as measured by the O:E ratio and/or c-statistic) of European-originated breast cancer risk prediction models validated in European populations.


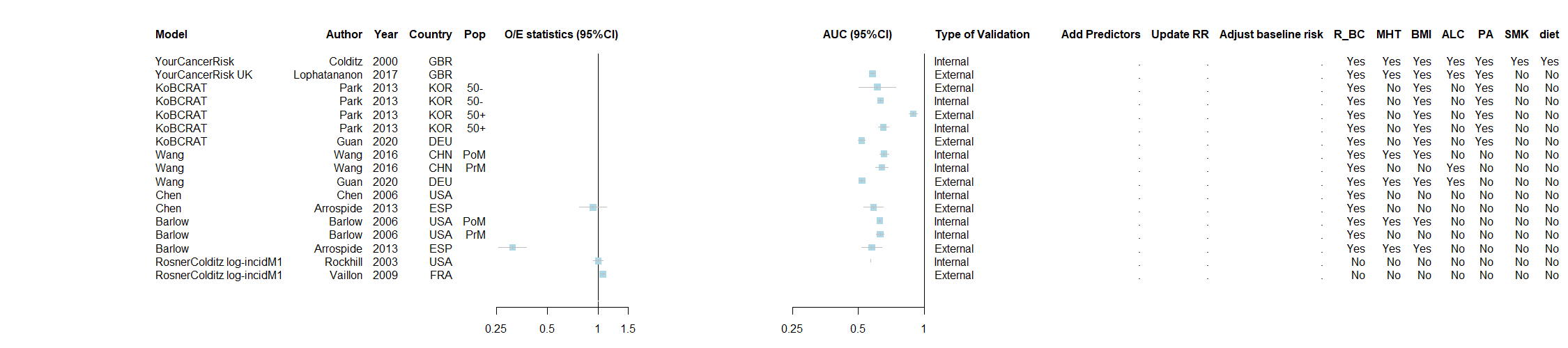


**Supplementary Figure 5** Predictive performance (as measured by the O:E ratio and/or c-statistic) of non-European-originated breast cancer risk prediction models validated in European populations.
