# Supplementary material for: Understanding the contribution of lifestyle in breast cancer risk prediction: a systematic review of models applicable to Europe
Source: BMC Cancer. 2023 Jul 21;23:687. doi: 10.1186/s12885-023-11174-w (PMC10360320; doi:10.1186/s12885-023-11174-w)
Supplement: Supplementary file 2 — Additional file 2. [file 12885_2023_11174_MOESM2_ESM.docx]

Figures and Tables

**SUPPLEMENTARY TABLE 1** Overview of studies describing risk prediction models for the incidence of primary breast cancer, applicable for European populations.

| **Author, year,  model name, if available**  **Country** | **Study setting** | **BC**  **incid time** | **Sample size:  Total N**  **(N cases)** | **Missing data handling  (%missing)** | **Model (variable selection)** | **Performance measures** | **PROBAST** | | | |
| --- | --- | --- | --- | --- | --- | --- | --- | --- | --- | --- |
|  |  |  |  |  |  |  | Pa | P | O | A |
| Amir et al., 2003 [1] Gail (M1) TC/IBIS (M2)  GBR | Family History Evaluation and Screening Programme (since 1987) | 10-y | V: 3,150 (64) | CC  (30.1%) | NA | E:O ratio  AUC |  |  |  |  |
| Tyrer, et al., 2004 [2] TC / IBIS (M1)  GBR | BC incidence rates taken from UK national statistics | 10-y | NA | NA | Cox (full model  defined *a priori* using segregation analyses and literature-based RR for non-familial, hormonal, hormonal and clinical RFs) | NI |  |  |  |  |
| Boyle et al., 2004 [3] M-Nutrient, <50/≥50 (M1, 2) M-Food, <50/≥50 (M3, 4) Gail (M5) Gail-ITA (M6)  ITA | D, U: Multicentre case-control study (1991-1994)  V: Italian Tamoxifen Trial (1992-1997) | 5-y | D, U: 5,157 (2,569)  V: 5,408 (79) | NI | M1-4: Logistic (full model defined a priori)  M5: Risk Tool  M6: Updated ORs, and ITA incidence and mortality rates | E:O ratio  AUC |  |  |  |  |
| Decarli et al., 2006 [4] Gail (M1) Gail-ITA (M2) Gail-ITA1 (M3)  ITA | U: ITA Multicentre Case-Control Study of Diet and Breast Cancer (1991-1994)  V: Florence-EPIC study (1993-1998) | 5-y | V: 10,031 (194)  U: 5,157 (2,569) | V: middle category imputation (<1%)  U: CC (0.2%) | M1: Risk Tool  M2, 3: Updated ORs with ordinal (M2) and categorical (M3) codes, and ITA incidence and mortality rates | E:O ratio  AUC |  |  |  |  |
| Novotny et al., 2006 [5] Gail (M1) Gail-CZE (M2) Gail-CZE + 4RFs (M3)  CZE | Case-control study with age-matched controls selected from a mammary care unit (2000-2004) | 5-y | V, U: 4,598 (2,299) | NI | M1: Risk Tool  M2, 3: Updated ORs with inclusion of 4RFs (M3), and CZE incidence and mortality rates | NI |  |  |  |  |
| Crispo et al., 2008 [6] Gail-ITA (M1) Gail-ITA + SDR (M2) Gail-ITA + F-SDR (M3)  ITA | Case-control study with BC cases and hospitalised controls recruited in the National Cancer Institute of Naples (1997-2000) | NI | V, U: 1,765 (558) | CC  (5.7%) | M1-3: Updated ORs with inclusion of SDR (M2) and F-SDR (M3) | AUC |  |  |  |  |
| Viallon et al., 2009 [7] Rosner Colditz (M1)  FRA | E3N cohort | 10-y | V: 80,883 (2,765) | CC  (12.1/%) | M1: Risk Tool | E:O ratio |  |  |  |  |
| Ulusoy et al., 2010 [8] Gail (M1)  Gail + B_FED (M2)  TUR | Case-control study with BC cases and controls from the mammography care unit (2002-2008) | 5-y | V, U: 1,290 (640) | CC  (NI) | M1: Risk Tool  M2: Estimated ORs with inclusion of B_FED | NI |  |  |  |  |
| Wacholder et al., 2010 [9] Gail (M1) Gail + PRS11 (M2)  POL | Polish Breast Cancer case-control Study (2000-2003) | 5-y | V, U: 2,692 (1,262) | CC  (17.4%) | M1, 2: Updated ORs for Gail RFs (M1) and literature-based individual SNPs (M2), and POL incidence and mortality rates | AUC |  |  |  |  |
| Petracci, et al., 2011 [10] 5NMRF + 2MRF (M1)  ITA | D: multi-centre case-control study from hospital medical records (1991-1994)  V: Florence EPIC (1998-2004) | 10-y | D: 5,027 (2,523)  V: 8,426 (206) | CC  (D: 2.5%; V: 16%) | Logistic (entry criteria of p<0.05 in multivariate) | E:O ratio  AUC |  |  |  |  |
| Darabi, et al., 2012 [11] Gail-SWE (M1) Gail-SWE + PMD + BMI (M2) Gail-SWE + PRS7/18 (M3, 4) Gail-SWE + PMD + BMI + PRS7/18 (M5, 6)  SWE | Swedish population-based case-control study of PoM women (1993-1995) | 5-y | M1,2: 3,411 (1,739);  M3, 4: 3,092 (1,566);  M5, 6: 1,873 (1,017) | CC  (0-45.1%) | M1-8: Updated ORs for BBD, NFDR_BC with offset for other Gail RFs, and published ORs for PMD (M2, 5, 6), BMI (M2, 5, 6), PRS (M3, 4, 5, 6), and SWE incidence and mortality rates | Plot  H-L test  AUC |  |  |  |  |
| Hüsing et al., 2012 [12] Covariate M (M1) M1 + PRS18 (M2)  USA, EUR | Nested case-control studies from BPC3 (1989-1998) | 5-y | D: 9,224 (4,006)  V: 4,612 (2,003) | MI  (1-21%) | M1, 2: logistic (backward with RF entry criteria of chosen in more than 5 of the 10 selected models (M1) and  literature-based individual SNPs (M2)) | AUC |  |  |  |  |
| Rauh et al., 2012 [13] RFs-M (M1) M1 + PMD (M2) M1 + DA (M3) M1 + PMD + DA (M4)  DEU | Bavarian Breast Cancer Cases and Control Studies (2002-2010) | NI | D, V: 992 (561) | CC  (15.2%) | M1-3: logistic (full model defined *a priori*) | AUC |  |  |  |  |
| Arrospide et al., 2013 [14] Gail (M1) Chen (M2) Barlow (M3)  ESP | Screening data of BC early-detection program in Sabadell-Cerdanyola aera (1995-1998) | 3-, and 5-y | V, M1: 13,446 (107)  V, M2: 12,435 (97)  V, M3: 13,709 (108) | CC  (0-9.3%) | M1, 2, 3: ESP incidence and mortality rates | H-L test  E:O ratio  AUC |  |  |  |  |
| Buron et al., 2013 [15] Gail  ESP | Screening data of Parc de Salut Mar (1996-2003) | 5-y | V: 1,662 (52) | CC (~75%) | Risk Tool | AUC |  |  |  |  |
| Pastor-Barrisuo et al., 2013 [16] Gail (M1) Gail-Navarre (M2) Gail-recalibrated (M3)  ESP | Navarre Breast Cancer Screening Program  (NBCSP) cohort (1996-1998) | 5-y | V, U: 54,649 (835) | CC (0.05%) | M1: Risk Tool  M2: Updated HRs by a piecewise exponential model with Gail RFs and ordinals codes  M3: Published original Gail RRs, and NBCSP incidence and mortality rates and RFs prevalence among cases | E:O ratio  AUC |  |  |  |  |
| Warwick et al., 2014 [17] TC (M1) TC + PMD_dr (M2)  GBR | Nested case-control study from the placebo arm of the IBIS-I (1992-2001) | 10-y | V, U: 558 (72) | CC (<2%) | M1: Risk Tool  M2: Logistic (fitted for PMD_dr and absolute TC risk score) | AUC |  |  |  |  |
| Brentnall et al., 2015 [18] TC (M1) TC + PMD_dr (M2) Gail (M3) Gail + PMD_dr (M4)  GBR | Cohort screening data from the PROCAS (2009-2013) | 10-y | 50,628 (697) | Mean value imputation for PMD_dr (7%) | M1, 3: Risk Tool  M2, 4: Logistic (fitted for PMD_dr and absolute TC or Gail risk score) | Plot  AUC |  |  |  |  |
| Dartois et al., 2015 [19] kNN-PrM (M1) kNN-PoM (M2) Cox-PrM (M3) Cox-PoM (M4)  Gail (M5)  FRA | E3N cohort (1990) | 5-y | D: PrM:22,969 (393); PoM: 27,992 (537)  V: PrM: 58,543 (78): PoM: 7,331 (98) | Modal category, median value imputation (<5%) | M1, 2: kNN (Euclidean distance with at least 20 neighbours)  M3, 4: Cox (stepwise forward by optimising AUC values)  M5: Risk Tool | Plot  E:O ratio  AUC |  |  |  |  |
| Hippisley-Cox, et al., 2015 [20] QCancer (M1)  GBR | QResearch Database of GP health records (1998-2003) | 10-y | D: 2,470,791 (40,251)  V: 814,578 (NS) | MI  (5%) | Cox (stepwise backward selection) | Plot  AUC |  |  |  |  |
| Vachon et al., 2015 [21] BCSC-M (M1) BCSC-PRS76 (M2)  DEU | Bavarian Breast Cancer Cases and Controls Studies (2002-2012) | 5-y | U: 879 (512) | CC  (19.1%) | M1: Risk Tool  M2: Updated by published ORs for PRS | AUC |  |  |  |  |
| Maas et al., 2016 [22] NM-MRFs (iCARE) (M1) M1 + PRS92 (M2)  EUR, AUS, USA | Nested case-control within the cohorts selected for genetic studies from the BPC3 (1976-1998) | 10-y | D, V: 37,033 (17,171) | MICE  (0-40%) | M1: Logistic (RFs defined *a priori)*  M2: Estimated ORs for 24 literature-based SNPs genotyped in BPC3 and published ORs for 68 SNPs | AUC |  |  |  |  |
| Cuzick et al., 2017 [23] TC (M1) TC + PRS88 (M2)  GBR | IBIS-I trial (1992-2001) and Marsden trial (1986-1996) | 10-y | V, U: 995 (359) | CC  (22%) | M1: Risk Tool  M2: Logistic (fitted for PRS and absolute TC risk score) | H-L test  AUC |  |  |  |  |
| Eriksson, et al., 2017 [24] MammoDetect-PrM/PoM (M1, 2) Gail (M3) TC (M4)  SWE | KARMA cohort (2011-2013) | 2-y | D, V:  PrM: 758 (152)  PoM: 1,407 (281) | CC  (24%) | M1, 2: Logistic (branch-and-bound Furnival and Wilson statistics)  M3, 4: Risk Tool | AUC |  |  |  |  |
| Evans, et al., 2017 [25] TC (M1) TC + PRS18 (M2)  GBR | Case-control study with BC cases and controls from the Manchester family-history clinic (1987-2014) | 10-y | U: 9,135 (489) | CC  (2%) | M1: Risk Tool  M2: Logistic (fitted for PRS and absolute TC risk score) | E:O ratio  AUC |  |  |  |  |
| Hüsing et al., 2017 [26] Gail (M1) Gail + TST, E2, SHBG, IGF-I-PrM/-PoM (M2, 3) Gail + TST-PrM (M4) Gail + TST, E2, SHBG, PoM (M5) Pfeiffer (M6) Pfeiffer + TST, E2, SHBG, IGF-I (M7) Pfeiffer + TST, E2, SHBG (M8)  DNK, FRA, DEU, GRE, ITA, NLD, ESP, GBR | EPIC breast cancer nested case-control study (1992-2002) | 5-y  10-y | V, U:  M1, 2, 3, 4, 5:  PrM: 1,114 (430)  PoM: 2,111 (878)  M6, 7, 8: PoM, >50y: 1,702 (661) | Gail: zero value imputation (53%)  Pfeiffer: CC (6%) | M1, 6: Risk Tool  M2-5, 7, 8: Estimated OR for BM with offset for absolute Gail or Pfeiffer risk score (backwards with BMs entry criteria of P<0.157 and AIC improved (M4, 5, 8)) | AUC |  |  |  |  |
| Lophatananon, et al., 2017 [27]YourCancerRisk-UK (M1)  GBR | UK Biobank study (2007-2010) with survey data from UK National surveys or EUR cohorts, BC incidence and mortality rates from Cancer Research UK, ONS | 10-y | U: 238,981 (3,378) | NI | Updated RRs for RFs, and updated population RF exposure prevalence and BC incidence and mortality | Plot  H-L test  E:O ratio  AUC |  |  |  |  |
| Dierssen-Sotos et al., 2018 [28] MRFs (M1) NMRFs (M2) M1+M2+PRS92 (M3)  ESP | MCC-Spain population-based multi-case-control study on common tumours (2008-2013) | NI | 3,642 (1,732) | NI | M1-3: Logistic (full model defined *a priori*) | AUC |  |  |  |  |
| Gabrielson et al., 2018 [29] PrM-Model (M1)  PoM-Model (M2) M1 + PRL (M3) M2 + PRL (M4) Gail (M5) Gail + PRL (M6) TC (M7) TC + PRL (M8) CAD2Y (M9) CAD2Y + PRL (M10)  SWE | A nested case-control study of the KARMA cohort (2011-2015) | M1-4: NI  M5-8: 5-y  M9-10:  2-y | D, V: M1: 552 (147); M2: 1,279 (303)  V, U: M5,6: PrM: 641 (233); PoM: 1,457 (476); M7, 8: PrM: 642 (233); PoM: 1,457 (476); M8, 9: PrM: 552 (147); PoM: 1,279 (303) | CC  (0.9-14.7%) | M1-4: Logistic (stepwise forward/ backward with entry criteria of P <0.15/ <0.2, and a linear term for natural log-transformed PRL (M3, 4))  M5, 7, 9: Risk Tool  M6, 8, 10: Logistic  (fitted for natural log-transformed PRL and absolute Gail, TC, CAD2Y risk score) | AUC |  |  |  |  |
| Li, et al., 2018 [30] Model ER+ (M1) Model ER- (M2) Model Omnibus (M3) Gail (M4)  NOR, SWE, DNK, GBR, NLD, DEU, FRA, ESP, ITA, GRE | D, V: EPIC (1992-2000) | 5-y | D, V: 281, 330 (ER+: 7,210; ER- 1,598; unknown 3,259) | MICE  (0-24%) | M1-3: Cause-specific piecewise-constant Cox (full model defined *a priori*) | Plot  E:O ratio  AUC |  |  |  |  |
| Lumachi et al., 2018 [31]  ESP | A case-control study using medical records of a cancer genetic clinic (2002-2006) | NI | 594 (297) | CC  (NI) | Logistic (stepwise forward) | AUC |  |  |  |  |
| Rudolph et al., 2018 [32] NM-MRFs (M1) M1 + PRS77 (M2)  EUR, AUS, USA | BCAC using for M1 (10 population-based (nested) case-control studies) (1990-2010) | NI | 58,684 (28,239)  M1: 13,168 (6,546) | CC  (M1: 1-67%; M2: NI) | M1: Logistic (full model defined *a priori*)  M2: Estimated ORs for literature-based SNPs | NI |  |  |  |  |
| van Veen et al., 2018 [33] TC (M1) TC + PMD_dr (M2) TC + PRS18 (M3) TC + PMD_dr + PRS18 (M4)  GBR | A case-cohort study of the PROCAS study (2009-2015) | 10-y | 9,363 (466) | CC (NI) | M1: Risk Tool  M2-4: Published ORs for PMD_dr and PRS with absolute TC score | Plot  E:O ratio  AUC |  |  |  |  |
| Clendenen et al., 2019 [34] Gail (M1) Gail-consortium (M2) Gail + AMH (M3) Gail + TST (M4) Gail + AMH + TST (M5)  GBR, ITA, SWE, USA | Nested case-control study in a consortium of 10 prospective cohorts | 5-y | 3,652 (1,762) | Lowest risk category imputation (~2%) | M1: Risk Tool  M2-5: Updated ORs for Gail RFs and estimated ORs for the BMs, summarised by RE-MA (M3, 4, 5), and country-specific incidence and mortality rates | AUC |  |  |  |  |
| Lee et al., 2019 [35] BOADICEA + RFs + PMD + PRS313 (M1)  GBR | NI | 10-y | NI | NI | Published RRs for RFs, PMD and PRS | NI |  |  |  |  |
| Usher-Smith et al., 2019 [36]  GBR | D: Quantitative literature review on cancer risk assessment based on European Code against Cancer 4^th^ edition with HSE (2005) and NDNS (2008-2012) survey data, and ONS mortality rates  V: EPIC-Norfolk (1993-1997) | 10-y | V: 12,828 (367) | NS | Expert Group consensus (classified convincing and probable RFs to be included in risk score) | Plot  AUC |  |  |  |  |
| Brentnall et al., 2020 [37] TC (M1) TC + PMD_rd (M2) TC + PRS143 (M3) TC + PMD_rd + PRS143 (M4)  GBR | A nested case-control of the PROCAS study (2009-2015) | 10-y | V: 2,073 (405) | CC  (0-4%) | M1: Risk Tool  M2-4: Published ORs for PMD_dr and PRS | E:O ratio  AUC |  |  |  |  |
| Pal Choudhury et al., 2020 [38] iCARE-BPC3 (M1) iCARE-Lit, <50 (M2) iCARE-Lit, ≥50 (M3) BCRAT (M4) TC (M5)  GBR | UK-based Generation Study (2003-2012) | 5-y | V, U: <50y: 28,232 (265)  ≥50y: 36,642 (598) | Hot deck imputation (NI) | M1: Published RRs taken from Maas et al., 2016  M2, 3: Literature-based RRs  M4, 5: Risk Tool | Plot  E:O ratio  AUC |  |  |  |  |
| Eriksson, et al., 2020 [39] KARMA-PrM/-PoM (M1, 2) M1/2 + RF-PrM/PoM (M3, 4) M3/4 + PRS-PrM/PoM (M5, 6) Gail (M7) Gail + PMD_dr (M8) TC (M9) TC + PMD_dr (M10)  SWE | D: Case-cohort of the KARMA cohort study (2011-2013)  V: MBTST (2010-2015), CSAW (2008-2015), KARMA (2011-2013) cohort study | M1-6: 2-y  M7-12: 5-y | D, M1-6: PrM: 4,610 (301); PoM: 5,067 (673)  V, M1, 2: 9,849 (104) from MBTST; 9,102 (613) from CSAW; 9,670 (179) from KARMA  V, M7-10: 10,350 (974) from KARMA | NS | M1-6: Logistic (full model defined *a priori*)  M7, 9: Risk Tool  M8, 10: Published ORs for PMD_dr | H-L test  AUC |  |  |  |  |
| Gabrielson, et al. 2020 [40] Gail (M1) Gail-DHEA/ DHEAS/ PMD/ PMD-DHEA/ PMD-DHEAS (M2, 3, 4, 5, 6) TC (M7) TC-DHEA/ DHEAS/ PMD/ PMD-DHEA/ PMD-DHEAS (M8, 9, 10, 11, 12) CAD2Y (M13) CAD2Y-DHEA/ DHEAS/ PMD/ PMD-DHEA/ PMD-DHEAS (M14, 15, 16, 17, 18)  SWE | Nested case-control study within the KARMA cohort (2011-2015) | M1-12: 5-y  M13-18: 2-y | V, U: PrM: 603 (222)  PoM: 1,249 (407) | CC  (0.3-38.4%) | M1, 7, 13: Risk Tool  M2-6, 8-12, 14-18: Logistic (fitted for natural log-transformed hormones, square root–transformed PMD to absolute Gail, TC, CAD2Y risk score) | AUC |  |  |  |  |
| Guan, et al., 2020 [41] Gail (M1) Novotny (M2) Park (M3) Wang (M4) Maas (M5) Rudolph (M6) Dierssen-Sotos (M7) M7 + MRS (M8) M7 + PRS (M9) M7 + MRS + PRS (M10)  DEU | Nested case-control study within the ESTHER study (2000-2002) | 14-y | 364 (101) | Mean value imputation (<5%) | M1-7: Risk Tool  M8-10: Published MRS and PRS added to risk score of M7 | H-L test  AUC |  |  |  |  |
| Hüsing et al., 2020 [42] BCRAT (M1) BCRAT-DEU (M2) BCRmod (M3) BCRmod-DEU (M4)  DEU | EPIC cohort of Heidelberg and Potsdam (1994-1998)  Population-based DEGS survey (2008-2011) | 5y  lifetime  1y | V, U: EPIC: 22,098 (256)  DEGS: 3,705 | MICE  (<20-100%) | M1, 3: Risk Tool  M2, 4: DEU incidence and mortality rates | Plot  H-L test  E:O ratio  AUC |  |  |  |  |
| Lakeman et al., 2020 [43] BOADICEA + RFs (M1) BOADICEA + RFs + PRS313 (M2)  NLD | Rotterdam Study-I and -II cohorts (1989 and 2000) | 10-y | 4,377 (163) | CC (Missing variables kept as missing) | M1, 2: Risk Tool taken from Lee et al., 2019 | Plot  AUC |  |  |  |  |
| Triviño et al., 2020 [44]  ESP | Case-control study from hospital and community screening medical records (2017-2018) | 5-y | D, V: 1,097 (455) | CC (27.2%) | Logistic (uni- prior to multivariate) | H-L test  AUC |  |  |  |  |
| Bonnet et al., 2021 [45]  FRA | Retrospective cohort study of BC screening centrer (CRCDC-OC) | 12-y | D: 2,768 (527) | CC (10.0%) | Cox (uni- prior to multivariate) |  |  |  |  |  |
| Pal Choudhury et al., 2021 [46] BOADICEA + PRS313 + RFs (M1) TC (M2) TC + PRS313 (M3)  GBR | Nested case-control study within the GS (2003-2012) | 5-y | V, U: 1,337 (619) | CC  (kept missing) | M1: Risk Tool taken from Lee et al., 2019  M2: Risk Tool  M3: Published ORs for PRS | Plot  E:O ratio  AUC |  |  |  |  |
| Hurson et al., 2021 [47] iCARE-BPC3 (M1) iCARE-BPC3 + PRS313 (M2) iCARE-Lit, <50/≥50 (M3, 4) iCARE-Lit, <50/≥50 + PRS313 (M5,6)  DEU, NLD, GBR, SWE | Nested cohorts from EPIC (DEU: 1994-1998, NLD: 1993-1997, GBR: 1993-2000), GS (GBR, 2003-2011), KARMA (SWE, 2011-2013), PROCAS (GBR, 2009-2013), and cohort from UK Biobank (GBR, 20016-2010) | 5-y | V, U: 181,321 (3,314) | At heck imputation | M1: Published RRs from Maas et al, 2016)  M2, 5, 6: Published ORs for PRS  M3-6: Literature-based RRs | Plot  E:O ratio  AUC |  |  |  |  |
| Louro et al., 2021 [48]  ESP | Spanish Breast Cancer Screening Programme of Barcelona (1995-2015) | 2- to 20-y | D: 73,181 (1,235)  V: 48,788 (823) | CC  (0.4%) | Cox (full model  defined *a priori*) | E:O ratio  AUC |  |  |  |  |
| Yiangou et al., 2021 [49]  CYP | MASTOS, a population-based case-control study (2004-2006) | 5-y | D, V: 1,780 (880) | CC  (22.1%) | Logistic (full model defined a priori) | H-L test  AUC |  |  |  |  |

Abbreviations: AIC, Akaike information criterion; AMH, anti-Müllerian hormone, a biomarker of ovarian reserve; AUC, area under the Receiver Operating Characteristic curve; AUS, Australia; BOADICEA, Breast and Ovarian Analysis of Disease Incidence and Carrier Estimation Algorithm; BBD, benign breast disease; BC, breast cancer; BCAC, Breast Cancer Association Consortium; BCRAT, Breast Cancer Risk Assessment Tool (Gail); BCSC-M, Breast Cancer Surveillance Consortium Model; BM, biomarkers; BMI, body mass index; BPC3, Breast and Prostate Cancer Cohort Consortium; B_FED, breast feeding; CAD2Y, short-term risk individual risk prediction of breast cancer; CC, complete-case-analyses; CSAW, Cohort of Screen-Age Women study in Sweden; CYP, Cyprus; CZE, Czech Republic; D, development; DA, absolute dense area of the breast; DEU, Germany; DHEA, dehydroepiandrosterone; DHEAS, dehydroepiandrosterone sulphate; DNK, Denmark; ESP, Spain; EPIC, European Prospective Investigation into Cancer and Nutrition study; EUR, Europe; E2, oestradiol; E3N cohort, Etude Epidemiologique aupres des femmes de la Mutuelle Generale de l’Education Nationale cohort; E:O ratio, expected to observed ratio; ER+/-, oestrogen receptor-positive/-negative breast cancer; FDR, first-degree relatives; FRA, France; GBR, Great Britain (UK); GRE, Greece; GS, Generation Study; HSE, Health Survey England; H-L test, Hosmer Lemeshow test; IBIS-I, International Breast Cancer Intervention Study, a randomised prevention trial; IGF-I, insulin-like growth factor I; ITA, Italy; KARMA, Karolinska Mammography Project for Risk Prediction of Breast Cancer cohort; kNN, K-nearest-neighbour algorithm; M, model; MBTST, Malmö Breast Tomosynthesis Screening Trial; MI, multiple imputation; MICE, multiple imputations by chained equations; MRS, methylation risk score, i.e. constructed as using hypermethylated CpGs methylation levels; NA, not applicable; NDNS, The National Diet and Nutrition Survey in UK; NFDR_BC, number of first-degree relatives with breast cancer; NI, no information; NLD, the Netherlands; NM_MRFs, non-modifiable and modifiable risk factors; NOR, Norway; ONS, Office of National Statistics in UK; OR, odds ratio; PMD, percentage mammographic density; PMD_dr, percentage mammographic density adjusted for age and BMI (i.e., the density residual in linear regression); POL, Poland; PoM, Post-Menopausal women; PROBAST, Prediction model Risk Of Bias Assessment Tool, including evaluation on the domain of Participants (P), Predictors (P), Outcome (O) and Statistical Analyses (A); PRL, prolactin; PrM, Pre-Menopausal women; PRS, polygenic risk score (genetic risk score, the sum across m number of SNPs (single-nucleotide polymorphisms) with risk-increasing alleles weighted by their weights); PROCAS, Predicting Risk of Breast Cancer At Screening case-control study; RE-MA, random effect meta-analyses; RF, risk factor; RR, relative risk; SDR, second-degree relatives; SWE, Sweden; TC, Tyrer-Cuzick risk model (or IBIS risk tool); TST, testosterone; TUR, Turkey; SHBG, sex hormone-binding globulin; U, update; USA, United States of America; V, validation

**SUPPLEMENTARY TABLE 2** Detailed overview of the predictors included in the final models of the 33 identified breast cancer risk prediction models developed for European populations.

| **Author, year, Model name, if applicable**  **Country**  **No of models** | **Demo** | **Medical history** | | | | **Lifestyle** | |
| --- | --- | --- | --- | --- | --- | --- | --- |
|  |  | **Family** | **Personal** | | |  |  |
|  |  |  | **Genetic** | **Reproductive and hormonal factors** | **Pre-existing (breast) disease** | **Anthropo-metrics** | **Lifestyle** |
| Tyrer, et al., 2004 [2] TC / IBIS (M1)  GBR | Age (c) | NFDR_BC | BRCA1/2  Low penetrance gene | AGEFLB (5-cat)  AGEMENA (x-cat)  AGEMENO (6-cat)  PAR (2-cat) | BBD (AH; 2-cat)  LCIS (2-cat) | BMI (5-cat)  Height (3-cat) |  |
| Boyle et al., 2004 [3] M-Nutrient, <50y (M1) M-Nutrient, ≥50y (M2) M-Food, <50 (M3) M-Food, ≥50y (M4)  ITA |  | NFDR_BC (2-cat)  R40_BC (2-cat) |  | AGEFLB (4-cat)  AGEMENA (3-cat)  AGEMENA*AGEFLB (M1, M3)  MHT (2-cat; M2, 4) |  | BMI (3-cat; M2, 4)  BMI*DS (3*2-cat; M2) | ALC (3-cat)  DS (5-cat): Beta-car & Vit E (M1, 2); F&V (M3, 4)  PA_OCC (3-cat; M2, 4) |
| Petracci, et al., 2011 [10] 5NMRF + 2MRF (M1)  ITA | Age (c)  Edu (3-cat) | NFDR_BC (2-cat) |  | AGEFLB (4-cat)  AGEMENA (3-cat) | NBIOP (2-cat) | BMI*Age50 (3*2-cat) | ALC (3-cat)  PA_LEI (2-cat)  PA_OCC (3-at) |
| Hüsing et al., 2012 [12] Covariate M (M1)  EUR, USA |  |  |  | AGEFLB (4-cat)  AGEMENA (3-cat)  AGEMENO (3-cat)  MHT (3-cat)  MS (3-cat)  NFTP (c) |  | BMI (c)  BMI*MS (c*3-cat) | ALC (3-cat)  SMK (3-cat) |
| Rauh et al., 2012 [13] RFs M (M1)  DEU | Age (c) |  |  | MS+MHT (3-cat)  PAR (x-cat) |  | BMI (c) |  |
| Dartois, et al, 2015 [19] kNN-PrM (M1) kNN-PoM (M2) Cox-PrM (M3) Cox-PoM (M4)  FRA | Age (6-cat) | NFDR_BC (3-cat) |  | AGEMENA (3-cat; M1)  AGEMENO (4-cat; M2, M4)  AGEMENO*Age (4*6-cat; M4)  MHT (5-cat; M2, M4)  PAR (5-cat; M4) | BBD (2-cat)  BBD*AGEMENO (2*4-cat; M4)  BBD*NFDR_BC* (2*3-cat; M3) | BMI (6-cat; M4) | ALC (6-cat; M4) |
| Hippisley-Cox, et al., 2015 [20] QCancer (M1)  GBR | Ethnicity (9-cat) | R_BC (2-cat) |  | MHT (2-cat)  OC (2-cat) | BBD (2-cat)  Depression (2-cat)  Prior C (lung, blood, ovarian; each 2-cat) |  | ALC (6-cat) |
| Maas et al., 2016 [22] NM-MRFs/iCARE (M1)  EUR, AUS, USA |  | FDR_BC (2-cat) |  | AGEFLB (9-cat)  AGEMENA (7-cat)  AGEMENO (10-cat)  MHT (6-cat)  PAR (5-cat) |  | BMI-PoM*MHT (10*2-cat)  Height (10-cat) | ALC (8-cat)  SMK (2-cat) |
| Eriksson, et al., 2017 [24] MammoDetect-PrM/PoM (M1, 2)  SWE |  | FDR_BC (2-cat) |  | MHT (2-cat) | MC_DIFF (c)  PMD (c)  PMD*MASS_N (c*c)  PMD_DIFF (c) | BMI (c) |  |
| Dierssen-Sotos et al., 2018 [28] MRFs (M1) NMRFs (M2)  ESP | Age (c; M2) | FDR_BC (2-cat; M2) |  | AGEFLB (9-cat; M2)  AGEMENA (7-cat; M2)  AGEMENO (10-cat; M2)  MHT (3-cat; M1)  MS (2-cat; M2)  PAR (5-cat; M2) |  | BMI (10-cat; M1)  Height (10-cat; M2) | ALC (8-cat; M1) |
| Gabrielson et al., 2018 [29]  PrM-M (M1)  PoM-M (M2)  SWE |  | R_BC (2-cat) |  | AGEMENA (c; M1)  MHT (2-cat; M2) | BBD (2-cat)  MASS_N (c; M2)  MASS_DIFF (c; M2)  MC_DIFF (c; M1)  MC_N (c)  PMD (c) | BMI (c; M2) | SMK (3-cat; M2) |
| Li et al., 2018 [30] Model ER+ (M1) Model ER- (M2) Omnibus (M3)  NOR, SWE, DNK, GBR, NLD, DEU, FRA, ESP, ITA, GRE |  |  |  | AGEFLB (5-cat)  AGEMENA (5-cat)  AGEMENO (4-cat)  B_FED (4-cat)  FTP (2-cat)  MHT (5-cat)  MS (2-cat)  PAR (3-cat) |  | BMI (4-cat)  BMI*MS (4*2-cat)  Height (c) | ALC (4-cat) |
| Lumachi et al, 2018 [31]  ESP | Age (2-cat) |  |  | MHT (2-cat)  MMI (2-cat) | BMD (2-cat) |  |  |
| Rudolph et al., 2018 [32] NM-MRFS (M1)  EUR, AUS, USA |  |  |  | AGEFTP (4-cat)  AGEMENA (7-cat)  MHT (2-cat)  PAR (2-cat) |  | BMI (4-cat)  Height (5-cat) | ALC (7-cat) |
| Lee et al., 2019 [35] Extended BOADICEA  GBR | Age (c)  Ashkenazi Jewish origin (2-cat) | R_BOPmBPaC (2-cat) | BRCA1/2  PALB2  CHECK2  ATM  PRS313 | AGEFTP (4-cat)  AGEMENA (7-cat)  AGEMENO (5-cat)  MHT (4-cat)  OC (3-cat)  PAR (4-cat) | BBD (5 B tumour pathologies; 2-cat)  PMD (BI-RADS, 4-cat) | BMI (4-cat)  Height (5-cat) | ALC (7-cat) |
| Usher-Smith et al., 2019 [36]  GBR |  |  |  |  |  | BMI (c; by MS) | ALC (c)  PA (c) |
| Eriksson, et al., 2020 [39] KARMA+RFs-PrM (M1) KARMA+RFs-PoM (M2)  SWE |  | FDR_BC (2-cat) |  | MHT (2-cat; M2) | PMD_dr (c)  MC_P (c)  MASS_P (c)  PMD_DIFF (c)  MC_P_DIFF (c) MASS_P_DIFF(c) | BMI (c) | ALC (2-cat)  SMK (2-cat) |
| Triviño et al., 2020 [44]  ESP | Age (8-cat) | NFSDR_BC (7-cat) | PRS92 (c) | AGEFLB (6-cat)  AGEMENA (6-cat)  AGEMENO (5-cat)  MS*Age (2*8-cat) | PMD (5-cat)  PMD*Age (5*8-cat- |  |  |
| Bonnet et al., 2021 [45]  FRA | Age (c)  Financial difficulties/ no vacation (2-cat) | FDR_BC (mother only; 2-cat) |  | AGEMENA (2-cat)  AGEMENO (2-cat)  AGEMENA*AGEMENO (2*2-cat) | PMD (BI-RADS; 4-cat) |  |  |
| Louro et al., 2021 [48]  ESP | Age (4-cat) | FDR_BC (2-cat) |  |  | BBD (4-cat)  MMG (6-cat) |  |  |
| Yiangou et al., 2021 [49]  CYP | Age (4-cat) | FDR_BC (2-cat) | PRS15 (c) | AGEFLB (5-cat)  AGEMENA (3-cat)  B_FED (2-cat)  MHT (2-cat)  MS (2-cat)  PAR (2-cat) |  | BMI (4-cat)  Height (c) | SMK (2-cat) |

Abbreviations: AGEFLB, age at first living childbirth; AGEFTP, age at first full-term pregnancy; AGEMENA, age at menarche; AGEMENO, age at menopause; ALC, alcohol consumption; AUS, Australia; BBD (AH), Breast Benign Disease (atypical hyperplasia); BC, breast cancer; BI-RADS, Breast Imaging Reporting and Data System; BMD, bone mineral density; BMI, body mass index; BOPmBPaC, breast, ovarian, prostate, male breast, pancreatic cancer; BRCA1/2, Breast Cancer gene 1 and 2; B_FED, breast feeding; C cancer; CYP, Cyprus; DEU, Germany; DNK, Denmark; DS, diet score; Edu, educational level; ER+/ER-, oestrogen receptor-positive/-negative breast cancer; ESP, Spain; EUR, Europe; FDR_BC, first-degree with breast cancer; FRA, France; F&V, fruit and vegetables; GBR, Great Britain (UK); GRE, Greece; ITA, Italy; kNN, k-nearest-neighbour algorithm; LCIS, Lobular carcinoma in situ; M, model; MASS_DIFF, absolute differences between right and left breasts in the number of masses; MASS_N, number of masses (a lump in the breast caused by abnormal growth of cells, a cyst, hormonal changes or an immune reaction); MASS_P_DIFF, percentage differences between right and left breasts in the number of masses; MC_DIFF, absolute differences between right and left breasts in the number of microcalcifications; MC_N, number of microcalcifications (tiny deposits of calcium in breast soft tissue); MC_P, probability of malignancy for the suspicious microcalcifications with malignant potential using the maximum of left and right breast views; MC_P_DIFF, percentage differences between right and left breasts in the number of microcalcifications; MHT, menopausal hormone therapy (hormonal replacement therapy); MMG, mammography features including masses, calcifications, asymmetric density, architectural distortion or multiple of these; MMI, interval between menarche and menopause; MRF, modifiable risk factors; MS, menopausal status; NBIOP, number of previous breast biopsies; NFDR_BC, number of first-degree relatives with breast cancer (mother, sister, daughter); NFTP, the number of full-term pregnancies; NF-SDR_BC, number of first- and second-degree relatives with breast cancer; NMRF, non-modifiable risk factors; NOR, Norway; NSDR_BC, number of second-degree relatives with breast cancer (aunts, grandmothers); NR_BC, number of relatives with breast cancer; OC, oral contraceptive use; PA, physical activity; PA_LEI, leisure-time physical activity; PA_OCC, occupational physical activity; PAR, parity (the number of live births); PMD, percentage mammographic density; PMD_dr, percentage mammographic density adjusted for age and BMI (i.e., the density residual in linear regression); PoM, post-menopausal women; PrM, pre-menopausal women; PRS, polygenic risk score (genetic risk score, the sum across m number of SNPs (single-nucleotide polymorphisms) with risk-increasing alleles weighted by their weights); RFs, risk factors; R_BC, relatives with breast cancer; R40_BC, age of the relative with breast cancer using 40 as cut-off; SMK, smoking; SWE, Sweden; TC, Tyrer-Cuzick risk model (or IBIS risk tool); USA, United States of America; (c), continuous variable; (x-cat), categorical variable with x categories

**SUPPLEMENTARY TABLE 3** Detailed overview of the existing models for the incidence of primary breast cancer, that are validated and/or updated for European populations, including their original predictors and modifications to existing models.

|  | **Variables** | **Model validation studies in European populations** | **Modifications to existing models** | |  |
| --- | --- | --- | --- | --- | --- |
|  |  |  | **Inclusion of additional variables** | **Update of coefficients (relative risks)** | **Adjustment of baseline risk/hazard** |
| **Models developed in EUR population** | | | | | |
| IBIS Risk Assessment Calculator  V6  Tyrer et al., 2004 [2]  GBR | Age, AGEMENA, PAR, AGEFLB, MS, AGEMENO, height, weight, MHT (including length of use), BBD (H, AH, LCIS), OvC, FDR_BC (OvC, bilaBC, BC, Age; mother, sisters, paternal gran, maternal gran, paternal aunts, maternal aunts, daughters), SDR_BC (OvC, BC, Age; half-sisters, affected cousins, affected nieces), Genetic testing, Ashkenazi inheritance | Amir et al., 2003 [1]; Warwick et al., 2014 [17]; Brentnall et al., 2015 [18]; Eriksson et al., 2017 [24]; Gabrielson et al., 2018 [29]; van Veen et al., 2018 [33]; Brentnall et al., 2020 [37]; Eriksson et al., 2020 [39] | PMD: Warwick et al., 2014 [17]; Brentnall et al., 2015 [18]; van Veen et al., 2018 [33]; Brentnall et al., 2020 [37]; Eriksson et al., 2020 [39]; Gabrielson et al., 2020 [40]  BM: Gabrielson et al., 2018 [29]; Gabrielson et al., 2020 [40]  PRS: Cuzick et al., 2017 [23]; Evans et al., 2017 [25]; van Veen et al., 2018 [33]; Brentnall et al., 2020 [37]; Pal Choudhury et al., 2021 [46] | Additional: Warwick et al., 2014 [17]; Brentnall et al., 2015 [18]; Cuzick et al., 2017 [23]; Evans et al., 2017 [25]; Gabrielson et al., 2018 [29] | NA |
| V7 | + male relatives, competing mortality with GBR and SWE rates | Cuzick et al., 2017 [23]; Evans et al., 2017 [25]; Gabrielson et al., 2020 [40] |  |  |  |
| V8 | + PMD (%Volpara, %VAS, BI-RADS), competing mortality with SVN rates | Pal Choudhury et al., 2020 [38]; Pal Choudhury et al., 2021 [46] |  |  |  |
| BOADICEA  Antoniou et al., 2008 [50]  GBR | Underlying genotype (using BRCA1/2, PALB2, CHEK2 and ATM) | Lee et al., 2019 [35]; Lakeman et al., 2020 [43]; Pal Choudhury et al., 2021 [46] | RFs: Lee et al., 2019 [35]; Lakeman et al., 2020 [43]; Pal Choudhury et al., 2021 [46]  PMD: Lee et al., 2019 [35]  PRS: Lee et al., 2019 [35]; Lakeman et al., 2020 [43]; Pal Choudhury et al., 2021 [46] | NA | NA |
| Covariate M  Hüsing et al., 2012 [12]  EUR, USA | MS (3-cat); AGEMENO (3-cat); AGEFLB (4-cat); NFTP (c); AGEMNA (3-cat); MHT (3-cat); BMI (c); BMI*MS (c*3-cat); ALC (3-cat); SMK (3-cat) | Hüsing et al., 2012 [12] | PRS: Hüsing et al., 2012 [12] | NA | NA |
| RF-M  Rauh et al., 2012 [13]  DEU | Age (c); MS+MHT (3-cat); PAR (x-cat); BMI (c) | Rauh et al., 2012 [13] | PMD: Rauh et al., 2012 [13]  DA: Rauh et al., 2012 [13] | NA | NA |
| iCARE M  Maas et al., 2016 [22]  EUR, AUS, USA | AGEMENA (6-cat), AGEMENO (10-cat), AGEFLB (9-cat), PAR (5-cat), ALC (8-cat), height (10-cat), BMI (10-cat), MHT(3-cat), SMK (2-cat), FDR_BC (2-cat) | Maas et al., 2016 [22]; Guan et al., 2020 [41]; Pal Choudhury et al., 2020 [38]; Hürson et al., 2021 [47] | PRS: Maas et al., 2016 [22]  OC: Pal Choudhury et al., 2020 [38]; Hürson et al., 2021 [47] | Original: Pal Choudhury et al., 2020 [38]; Hürson et al., 2021 [47] | Pal Choudhury et al., 2020 [38]; Hürson et al., 2021 [47] |
| CADY2Y  Eriksson et al., 2017 [24]  SWE | Age (c), BMI (c), MHT (c), FDR_BC (2-cat), PMD (c), PMD_DIFF (c), MC_DIFF (c), PMD*MASS_N (c*c) | Gabrielson et al., 2018 [29]; Gabrielson et al., 2020 [40] | PMD: Gabrielson et al., 2020 [40]  BM: Gabrielson et al., 2018 [29]; Gabrielson et al., 2020 [40] | Additional: Gabrielson et al., 2018 [29] | NA |
| Dierssen-Sotos, 2018 [28]  ESP | Height (10-cat), BMI (10-cat), ALC (8-cat), AGEMENA (6-cat), AGEMENO (10-cat), AGEFLB (9-cat), PAR (5-cat), R_BC (2-cat), MS (2-cat), MHT (2-cat) | Dierssen-Sotos et al., 2018 [28]; Guan et al., 2020 [41] | MRS: Guan et al., 2020 [41]  PRS: Dierssen-Sotos et al., 2018 [28]; Guan et al., 2020 [41] | NA | NA |
| PrM/PoM-M  Gabrielson et al., 2018 [29]  SWE | PrM: R_BC (2-cat); AGEMENA (c); BBD (2-cat); PMD (c); MC_N (c); MC_DIFF (c)  PoM: R_BC (2-cat); MHT (2-cat); BBD (2-cat); PMD (c); MC_N (c); MASS_N (c); MASS_DIFF (c); BMI (c); SMK (3-cat) | Gabrielson et al., 2018 [29] | BM: Gabrielson et al., 2018 [29] | NA | NA |
| Rudolph et al., 2018 [32]  EUR, AUS, CAN, USA | AGEMENA (6-cat), PAR (2-cat), AGEFLB (4-cat), ALC (7-cat), Height (4-cat), BMI (4-cat), MHT (2-cat) | Rudolph et al., 2018 [32]; Guan et al., 2020 [41] | PRS: Rudolph et al., 2018 [32] | NA | NA |
| KARMA PrM/PoM + RFs  Eriksson et al., 2020 [39]  SWE | PrM: FDR_BC (2-cat); PMD_dr (c); MC_P (c); PMD_DIFF (c); MC_P_DIFF (c); MASS_P_DIFF (c); BMI (c); ALC (2-cat); SMK (2-cat)  PoM: FDR_BC (2-cat); MHT (2-cat); PMD_dr (c); MC_P (c); PMD_DIFF (c); MC_P_DIFF (c); MASS_P_DIFF (c); BMI (c); ALC (2-cat); SMK (2-cat) | Eriksson et al., 2020 [39] | PRS: Eriksson et al., 2020 [39] | NA | NA |
| **Models developed in a non-EUR population** | | | | | |
| Gail M  Original  Gail et al., 1989 [51]  USA | Age (2-cat), NFDR_BC (3-cat), AGEMENA (3-cat), AGEFLB (4-cat), NBIOP (3-cat), NBIOP*Age, AGEFLB*NFDR_BC | Boyle et al., 2004 [3]; Declari et al., 2006 [4]; Novotny et al., 2006 [5]; Crispo et al., 2008 [6]; Wacholder et al., 2010 [9]; Darabi et al., 2012 [11]; Arrospide et al., 2013 [14]; Pastor-Barrisuo et al., 2013 [16]; Guan et al., 2020 [41] | R_BC: Novotny et al., 2006 [5]; Crispo et al., 2008 [6]; Guan et al., 2020 [41]  B_INFLAM: Novotny et al., 2006 [5]  N_CON : Novotny et al., 2006 [5]; Guan et al., 2020 [41]  B_FED : Ulusoy et al., 2010 [8]  BMI : Novotny et al., 2006 [5]; Darabi et al., 2012 [11]; Guan et al., 2020 [41]  PMD : Darabi et al., 2012 [11]; Brentnall et al., 2015 [18]; Eriksson et al., 2020  [39]; Gabrielson et al., 2020 [40]  BM: Hüsing et al., 2017 [26]; Gabrielson et al., 2018 [29]; Gabrielson et al., 2020 [40]; Clendenen et al., 2019 [34]  PRS: Wacholder et al., 2010 [9]; Darabi et al., 2012 [11] | Original: Boyle et al., 2004 [3]; Declari et al., 2006 [4]; Novotny et al., 2006 [5]; Crispo et al., 2008 [6]; Wacholder et al., 2010 [9]; Pastor-Barrisuo et al., 2013 [16]; Clendenen et al., 2019 [34]; Hüsing et al., 2020 [42]  Additional: Ulusoy et al., 2010 [8]; Wacholder et al., 2010 [9]; Brentnall et al., 2015 [18]; Hüsing et al., 2017 [26]; Gabrielson et al., 2018 [29] | Boyle et al., 2004 [3]; Declari et al., 2006 [4]; Wacholder et al., 2010 [9]; Darabi et al., 2012 [11]; Arrospide et al., 2013 [14]; Pastor-Barrisuo et al., 2013 [16]; Clendenen et al., 2019 [34]; Hüsing et al, 2020 [42] |
| Updated  Gail et al., 2001 [52]  USA | + BBD (AH; 2-cat) | Amir et al., 2003 [1]; Ulusoy et al., 2010 [8]; Brentnall et al., 2015 [18]; Dartois et al., 2015 [19]; Eriksson et al., 2017 [24]; Hüsing et al., 2017 [26]; Gabrielson et al., 2018 [29]; Li et al., 2018 [30]; Eriksson et al., 2020 [39]; Gabrielson et al., 2020 [40] |  |  |  |
| NCI Breast Cancer Risk Assessment Tool (BCRAT)  Costantino et al., 1999 [53]; Gail et al., 2007 [54]; Matsuno et al., 2011 [55]; Banegas et al., 2016 [56]  USA | Age (2-cat), NBIOP (2-cat), BBD (H; 2-cat), AGEMENA(3-cat), AGEFLB (4-cat), NFDR_BC (3-cat), race (10-cat) | Buron et al., 2013 [15]; Clendenen et al., 2019 [34]; Hüsing et al., 2020 [42]; Pal Choudhury et al., 2020 [38] |  |  |  |
| BCRmod,  Pfeiffer et al., 2013 [57]  USA | Age (2-cat), NBIOP (2-cat), BBD (H; 2-cat), AGEMENA(3-cat), AGEFLB (3-cat), NFDR_BC (3-cat), race (10-cat), BMI (4-cat); ALC (3-cat), MHT (3-cat) | Hüsing et al., 2017 [26]; Hüsing et al., 2020 [42] |  |  |  |
| Rosner and Colditz log-incidence BC-M  Rosner and Colditz et al., 1996 [58] and 2000 [59]  USA | AGEMENA (c), MIN(age, AGEMENO)-AGEMENA (c), age-AGEMENO (PoM, c), AGEFLB-AGEMENA, birth index (c), birth index*(age-ageMENO) (PoM, c) | Viallon et al., 2009 [7] | NA | NA | NA |
| Your Disease Risk Online v8  Colditz et al., 2000 [60]; and webtool [61]  USA | Age (c), C (2-cat), FDR_BOPC (3-cat for BOP, BC in sister and BC in mother), BRCA1/2 or other high-risk gene mutations (3-cat), Jewish ethnicity (2-cat), height (c), weight (c), weight at 18 (c), body shape (5-cat), SMK (3-cat), ALC (6-cat), F&V (2-cat), PA_LEI (2-cat), AGEMENA (2-cat), PAR (4-cat), AGEFLB_35 (2-cat), B_FED (2-cat), MS (3-cat), MHT (4-cat), MHT type (4-cat), OC (2-cat), birth weight (2-cat), tamoxifen or raloxifene (2-cat), BBD (2-cat), PMD (3-cat) | Lophatananon et al., 2017 [27] | NA | Original: Lophatananon et al., 2017 [27] | Lophatananon et al., 2017 [27] |
| Barlow et al., 2006 [62]  USA | PrM: age (4-cat), BBD (3-cat), NFDR_BC (4-cat), PMD (BI-RADS, 5-cat)  PoM: age (8-cat), Hispanic (3-cat), race (6-cat), BMI (5-cat), AGEFLB (4-cat), BBD (3-cat), NFDR_BC (4-cat), MHT (3-cat), surgical MS (3-cat), MMG (3-cat), PMD (BI-RADS, 5-cat) | Arrospide et al., 2013 [14] | NA | NA | NA |
| Chen et al., 2006 [63]  USA | NFDR_BC (3-cat), AGEFLB (4-cat), NBIOP (3-cat), PMD (5-cat), weight (6-cat), AH (4-cat) | Arrospide et al., 2013 [14] | NA | NA | Arrospide et al., 2013 [14] |
| Breast Cancer Surveillance Consortium  Tice et al., 2008 [64]  USA | Age (8-cat), race (7-cat), PMD (BI-RADS, 4-cat), BBD (6-cat), FDR_BC (2-cat) | Vachon et al., 2015 [21] | PRS: Vachon et al., 2015 [21] | NA | NA |
| Park et al., 2013 [65]  KOR | R_BC (2-cat), AGEMENA (3-cat), MS (2-cat), AGEMENO (4-cat), PAR (2-cat), BMI (3-cat), OC (2-cat), PA_LEI (2-cat) | Guan et al., 2020 [41] | NA | NA | NA |
| Wang et al., 2016 [66]  CHN | PrM: Age (7-cat), PAR (4-cat), NFDR_BC (3-cat), ALC (2-cat), LAN (3-cat), sleep quality (4-cat)  PoM: Age (7-cat), BMI (4-cat), AGEMENA (6-cat), AGEFLB (3-cat), PAR, B_FED (4-cat), MHT (2-cat), NFDR_BC (3-cat), BBD (2-cat), LAN (3-cat), sleep quality (4-cat) | Guan et al., 2020 [41] | NA | NA | NA |

Abbreviations: AGEFLB, age at first living childbirth; AGEFTP, age at first full-term pregnancy; AGEMENA, age at menarche; AGEMENO, age at menopause; ALC, alcohol consumption; AUS, Australia; BBD (AH), Breast Benign Disease (atypical hyperplasia); BC, breast cancer; BCRAT, Breast Cancer Risk Assessment Tool (updated Gail model); BCRmod, Breast, Endometrial and Ovarian Risk Assessment; BI-RADS, Breast Imaging Reporting and Data System; BOADICEA, Breast and Ovarian Analysis of Disease Incidence and Carrier Estimation Algorithm; BM, biomarker; BMD, bone mineral density; BMI, body mass index; BOPC, Breast, Ovarian, Prostate Cancer; BRCA1/2, Breast Cancer gene 1 and 2; B_FED, breast feeding; B_INFL, breast inflammation; C cancer; CAD2Y, short-term risk individual risk prediction of breast cancer; CHN, China; CYP, Cyprus; DA, absolute dense area of the breast; DEU, Germany; DNK, Denmark; DS, diet score; Edu, educational level; ER+/ER-, oestrogen receptor-positive/-negative breast cancer; ESP, Spain; EUR, Europe; FDR_BC, first-degree with breast cancer; FRA, France; F&V, fruit and vegetables; GBR, Great Britain (UK); GRE, Greece; ITA, Italy; KOR; South Korea; kNN, k-nearest-neighbour algorithm; LAN, light at night; LCIS, Lobular carcinoma in situ; M, model; MASS_DIFF, absolute differences between right and left breasts in the number of masses; MASS_N, number of masses (a lump in the breast caused by abnormal growth of cells, a cyst, hormonal changes or an immune reaction); MASS_P_DIFF, percentage differences between right and left breasts in the number of masses; MC_DIFF, absolute differences between right and left breasts in the number of microcalcifications; MC_N, number of microcalcifications (tiny deposits of calcium in breast soft tissue); MC_P, probability of malignancy for the suspicious microcalcifications with malignant potential using the maximum of left and right breast views; MC_P_DIFF, percentage differences between right and left breasts in the number of microcalcifications; MHT, menopausal hormone therapy (hormonal replacement therapy); MMG, mammography features including masses, calcifications, asymmetric density, architectural distortion or multiple of these; MMI, interval between menarche and menopause; MRF, modifiable risk factors; MRS, methylation risk score, i.e. constructed as using hypermethylated CpGs methylation levels; MS, menopausal status; NBIOP, number of previous breast biopsies; NFDR_BC, number of first-degree relatives with breast cancer (mother, sister, daughter); NFTP, the number of full-term pregnancies; NF-SDR_BC, number of first- and second-degree relatives with breast cancer; NMRF, non-modifiable risk factors; NOR, Norway; NSDR_BC, number of second-degree relatives with breast cancer (aunts, grandmothers); NR_BC, number of relatives with breast cancer; N_CON, number of conceptions; OC, oral contraceptive use; PA, physical activity; PA_LEI, leisure-time physical activity; PA_OCC, occupational physical activity; PAR, parity (the number of live births); PMD, percentage mammographic density; PMD_dr, percentage mammographic density adjusted for age and BMI (i.e., the density residual in linear regression); PoM, post-menopausal women; PrM, pre-menopausal women; PRS, polygenic risk score (genetic risk score, the sum across m number of SNPs (single-nucleotide polymorphisms) with risk-increasing alleles weighted by their weights); RFs, risk factors; R_BC, relatives with breast cancer; R40_BC, age of the relative with breast cancer using 40 as cut-off; SMK, smoking; SVN, Slovenia; SWE, Sweden; TC, Tyrer-Cuzick risk model (or IBIS risk tool); USA, United States of America; VAS, visual analogue scale; Volpara, Volpara density grade, i.e., the volumetric breast density percentage of a mammogram; (c), continuous variable; (x-cat), categorical variable with x categories

**SUPPLEMENTARY TABLE 4** Published and transformed model-specific effect sizes of the commonly shared lifestyle risk factors employed in existing risk prediction models for breast cancer developed or validated in a European population.

A: BMI as predictor for premenopausal women

| **Author** | **Year** | **Country** | **ModelName** | **Population** | **Comparison** | **Model** | **Published ES** | **PoRo** | **Transformed RR** |
| --- | --- | --- | --- | --- | --- | --- | --- | --- | --- |
| Colditz | 2000 | USA | YourCancerRisk | PrM | Overweight | Lit | 0.80 | NA | 0.80 |
| Tyrer | 2004 | GBR | TC | PrM | Overweight | Lit | 1.08 (0.79;1.48) | NA | 1.08 (0.79;1.48) |
| Lophatananon | 2017 | GBR | YourCancerRisk UK | PrM | Overweight | NS | 0.80 (0.80;1.00) | NA | 0.80 (0.80;1.00) |
| Li | 2018 | EPIC | ER+/ER-/Omnibus | PrM | Overweight | CPhM | 0.99 (0.92;1.07) | 0.00 | 0.99 (0.92;1.07) |
| Lee | 2019 | GBR | Ext BOADICEA | 50- | Overweight | Lit | 0.92 (0.84; 1.01) | NA | 0.92 (0.84; 1.01) |
| Pal Choudhury | 2020 | GBR | iCARE-Lit | 50- | Overweight | Lit | 0.92 | NA | 0.92 |
| Rudolph | 2018 | EUR. AUS. USA | NM-MRFS |  | Overweight | LR | 1.11 (0.81;1.52) | 0.45 | 1.06 (0.89;1.23) |
| Pal Choudhury | 2020 | GBR | iCARE-BPC3 | PrM /  PoM-Never MHT | Overweight I | LR | 1.40 (1.04;1.87) | 0.01 | 1.39 (1.04;1.85) |
| Dierssen-Sotos | 2018 | ESP | MRFS |  | Overweight I | LR | 1.49 (1.10;2.03) | 0.46 | 1.21 (1.05;1.37) |
| Tyrer | 2004 | GBR | TC | PrM | Overweight II | Lit | 0.77 (0.47;1.28) | NA | 0.77 (0.47;1.28) |
| Pal Choudhury | 2020 | GBR | iCARE-BPC3 | PrM /  PoM-Never MHT | Overweight II | LR | 1.28 (0.93;1.76) | 0.01 | 1.28 (0.93;1.75) |
| Dierssen-Sotos | 2018 | ESP | MRFS |  | Overweight II | LR | 1.77 (1.29;2.43) | 0.46 | 1.30 (1.14;1.46) |
| Lee | 2019 | GBR | Ext BOADICEA | 50- | Obesity | Lit | 0.74 (0.66; 0.82) | NA | 0.74 (0.66; 0.82) |
| Pal Choudhury | 2020 | GBR | iCARE-Lit | 50- | Obesity | Lit | 0.74 | NA | 0.74 |
| Rudolph | 2018 | EUR. AUS. USA | NM-MRFS |  | Obesity | LR | 1.40 (1.01;1.95) | 0.45 | 1.19 (1.00;1.36) |
| Li | 2018 | EPIC | ER+/ER-/Omnibus | PrM | Obesity I | CPhM | 0.97 (0.85;1.10) | 0.00 | 0.97 (0.85;1.10) |
| Pal Choudhury | 2020 | GBR | iCARE-BPC3 | PrM /  PoM-Never MHT | Obesity I | LR | 1.58 (1.14;2.19) | 0.01 | 1.57 (1.14;2.17) |
| Dierssen-Sotos | 2018 | ESP | MRFS |  | Obesity I | LR | 1.36 (0.97;1.90) | 0.46 | 1.17 (0.98;1.34) |
| Li | 2018 | EPIC | ER+/ER-/Omnibus | PrM | Obesity II | CPhM | 1.12 (0.92;1.36) | 0.00 | 1.12 (0.92;1.36) |
| Pal Choudhury | 2020 | GBR | iCARE-BPC3 | P PrM /  PoM-Never MHT | Obesity II | LR | 1.45 (1.02;2.07) | 0.01 | 1.44 (1.02;2.05) |
| Dierssen-Sotos | 2018 | ESP | MRFS |  | Obesity II | LR | 1.40 (0.96;2.04) | 0.46 | 1.18 (0.98;1.38) |
| Novotny | 2006 | SZE | added to Gail M | | Ordinal | LR | 1.06 | 0.50 | 1.03 |
| Petraccio | 2011 | ITA | 5NMRF + 2MRF | 50- | Ordinal | LR | 1.26 (1.26;1.27) | 0.40 | 1.14 (1.14;1.14) |
| Hüsing | 2012 | GBR | Covariate M | PrM | Continuous | LR | 0.99 (0.97;1.01) | 0.43 | 0.99 (0.98;1.01) |
| Eriksson | 2017 | SWE | MammoDetectRS | PrM | Continuous | LR | 0.97 (0.93;1.02) | 0.20 | 0.98 (0.94;1.01) |
| Usher-Smith | 2019 | GBR |  | PrM | Continuous | Lit | 0.99 (0.98;0.99) | NA | 0.99 (0.98;0.99) |
| Eriksson | 2020 | SWE | KARMA+RF+PRS | PrM | Continuous | LR | 0.97 (0.95;1.00) | 0.07 | 0.97 (0.95;1.00) |
| Yiangou | 2021 | CYP |  |  | Continuous | LR | 1.02 (1.00;1.04) | 0.50 | 1.01 (1.00;1.02) |

B: BMI as predictor for postmenopausal women

| **Author** | **Year** | **Country** | **ModelName** | **Population** | **Comparison** | **Model** | **Published ES** | **PoRo** | **Transformed RR** |
| --- | --- | --- | --- | --- | --- | --- | --- | --- | --- |
| Colditz | 2000 | USA | YourCancerRisk | PoM | Overweight | Lit | 1.30 | NA | 1.30 |
| Tyrer | 2004 | GBR | TC | PoM | Overweight | Lit | 1.26 (1.09;1.47) | NA | 1.26 (1.09;1.47) |
| Barlow | 2006 | USA |  | PoM | Overweight | LR | 1.14 (1.07;1.23) | 0.01 | 1.14 (1.07;1.23) |
| Darabi | 2012 | SWE | added to Gail M | PoM | Overweight | Lit | 1.28 (0.90;1.80) | 0.49 | 1.13 (0.95;1.30) |
| Park | 2013 | KOR | KoBCRAT | 50+ | Overweight | LR | 1.16 (0.97;1.38) | 0.50 | 1.07 (0.98;1.16) |
| Lophatananon | 2017 | GBR | YourCancerRisk UK | PoM | Overweight | NS | 1.10 (1.10;1.20) | NA | 1.10 (1.10;1.20) |
| Li | 2018 | EPIC | ER+/ER-/Omnibus | PoM | Overweight | CPhM | 1.11 (1.04;1.18) | 0.00 | 1.11 (1.04;1.18) |
| Lee | 2019 | GBR | Ext BOADICEA | 50+ | Overweight | Lit | 1.13 | NA | 1.13 |
| Pal Choudhury | 2020 | GBR | iCARE-Lit | 50+ - Never MHT | Overweight | Lit | 1.13 | NA | 1.13 |
| Pal Choudhury | 2020 | GBR | iCARE-Lit | 50+ - MHT Type C | Overweight | Lit | 2.18 | NA | 2.18 |
| Pal Choudhury | 2020 | GBR | iCARE-Lit | 50+ - MHT Type E | Overweight | Lit | 1.49 | NA | 1.49 |
| Maas | 2016 | EUR. AUS. USA | NM-MRFS | PoM - Never MHT | Overweight I | LR | 1.21 (1.13;1.30) | 0.46 | 1.10 (1.07;1.14) |
| Maas | 2016 | EUR. AUS. USA | NM-MRFS | PoM - Ever MHT | Overweight I | LR | 1.06 (1.04;1.08) | 0.46 | 1.03 (1.02;1.04) |
| Pal Choudhury | 2020 | GBR | iCARE-BPC3 | PoM - Ever MHT | Overweight I | LR | 1.27 (0.98;1.63) | 0.01 | 1.27 (0.98;1.62) |
| Tyrer | 2004 | GBR | TC | PoM | Overweight II | Lit | 1.32 (1.07;1.57) | NA | 1.32 (1.07;1.57) |
| Maas | 2016 | EUR. AUS. USA | NM-MRFS | PoM - Never MHT | Overweight II | LR | 1.32 (1.20;1.44) | 0.46 | 1.15 (1.10;1.20) |
| Pal Choudhury | 2020 | GBR | iCARE-BPC3 | PoM - Ever MHT | Overweight II | LR | 1.12 (0.83;1.51) | 0.01 | 1.12 (0.83;1.50) |
| Barlow | 2006 | USA |  | PoM | Obesity | LR | 1.28 (1.17;1.40) | 0.01 | 1.28 (1.17;1.40) |
| Barlow | 2006 | USA |  | PoM | Obesity | LR | 1.47 (1.30;1.65) | 0.01 | 1.47 (1.30;1.64) |
| Darabi | 2012 | SWE | added to Gail M | PoM | Obesity | Lit | 1.67 (1.20;2.30) | 0.49 | 1.26 (1.09;1.41) |
| Park | 2013 | KOR | KoBCRAT | 50+ | Obesity | LR | 2.28 (1.49;3.48) | 0.50 | 1.39 (1.20;1.55) |
| Lee | 2019 | GBR | Ext BOADICEA | 50+ | Obesity | Lit | 1.25 | NA | 1.25 |
| Pal Choudhury | 2020 | GBR | iCARE-Lit | 50+ - Never MHT | Obesity | Lit | 1.25 | NA | 1.25 |
| Pal Choudhury | 2020 | GBR | iCARE-Lit | 50+ - MHT Type C | Obesity | Lit | 2.11 | NA | 2.11 |
| Pal Choudhury | 2020 | GBR | iCARE-Lit | 50+ - MHT Type E | Obesity | Lit | 1.45 | NA | 1.45 |
| Li | 2018 | EPIC | ER+/ER-/Omnibus | PoM | Obesity I | CPhM | 1.21 (1.10;1.34) | 0.00 | 1.21 (1.10;1.34) |
| Pal Choudhury | 2020 | GBR | iCARE-BPC3 | PoM - Ever MHT | Obesity I | LR | 1.27 (0.94;1.73) | 0.01 | 1.27 (0.94;1.72) |
| Maas | 2016 | EUR. AUS. USA | NM-MRFS | PoM - Never MHT | Obesity II | LR | 1.52 (1.38;1.68) | 0.46 | 1.22 (1.17;1.28) |
| Maas | 2016 | EUR. AUS. USA | NM-MRFS | PoM - Ever MHT | Obesity II | LR | 1.33 (1.27;1.40) | 0.46 | 1.15 (1.13;1.18) |
| Li | 2018 | EPIC | ER+/ER-/Omnibus | PoM | Obesity II | CPhM | 1.30 (1.11;1.53) | 0.00 | 1.30 (1.11;1.53) |
| Pal Choudhury | 2020 | GBR | iCARE-BPC3 | PoM - Ever MHT | Obesity II | LR | 1.29 (0.93;1.78) | 0.01 | 1.29 (0.93;1.77) |
| Boyle | 2004 | ITA | M-Nutrient | 50+ | Ordinal | LR | 0.98 (0.83;1.15) | 0.48 | 0.99 (0.90;1.07) |
| Boyle | 2004 | ITA | M-Food | 50+ | Ordinal | LR | 1.11 (1.02;1.21) | 0.48 | 1.05 (1.01;1.10) |
| Petraccio | 2011 | ITA | 5NMRF + 2MRF | 50+ | Ordinal | LR | 1.13 (1.13;1.13) | 0.48 | 1.07 (1.06;1.07) |
| Pfeiffer | 2013 | USA | BCRmod | 50+ | Ordinal | LR | 1.11 (1.07;1.16) | 0.00 | 1.11 (1.07;1.16) |
| Hüsing | 2020 | DEU | BCRmod | 50+ | Ordinal | LR | 1.00 (0.92;1.10) | 0.03 | 1.00 (0.92;1.10) |
| Hüsing | 2012 | GBR | Covariate M | PoM | Continuous | LR | 1.03 (1.00;1.05) | 0.43 | 1.02 (1.00;1.03) |
| Eriksson | 2017 | SWE | MammoDetectRS | PoM | Continuous | LR | 1.04 (1.01;1.06) | 0.20 | 1.03 (1.01;1.05) |
| Gabrielson | 2018 | SWE |  | PoM | Continuous | LR | 1.04 | 0.33 | 1.03 |
| Usher-Smith | 2019 | GBR |  | PoM | Continuous | Lit | 1.02 (1.02;1.03) | NA | 1.02 (1.02;1.03) |
| Eriksson | 2020 | SWE | KARMA+RF+PRS | PoM | Continuous | LR | 1.04 (1.02;1.06) | 0.13 | 1.03 (1.02;1.05) |

C: Alcohol consumption as predictor

| **Author** | **Year** | **Country** | **ModelName** | **Population** | **Comparison** | **Model** | **Published ES** | **PoRo** | **Transformed RR** |
| --- | --- | --- | --- | --- | --- | --- | --- | --- | --- |
| Hüsing | 2012 | GBR | Covariate M |  | Light | LR | 1.00 (0.91;1.11) | 0.43 | 1.00 (0.95;1.06) |
| Hippisley-Cox | 2015 | GBR | Qcancer |  | Light | CPhM | 1.11 (1.07;1.15) | 0.02 | 1.11 (1.07;1.15) |
| Maas | 2016 | EUR. AUS. USA | NM-MRFS |  | Light | LR | 1.00 (0.92;1.07) | 0.46 | 1.00 (0.96;1.04) |
| Dierssen-Sotos | 2018 | ESP | MRFS |  | Light | LR | 0.89 (0.71;1.12) | 0.47 | 0.94 (0.82;1.06) |
| Li | 2018 | EPIC | ER+/ER-/Omnibus | | Light | CPhM | 1.14 (1.05;1.24) | 0.00 | 1.14 (1.05;1.24) |
| Rudolph | 2018 | EUR. AUS. USA | NM-MRFs |  | Light | LR | 1.07 (0.93;1.22) | 0.49 | 1.03 (0.96;1.10) |
| Lee | 2019 | GBR | Ext BOADICEA | | Light | Lit | 1.03 (1.00; 1.06) | NA | 1.03 (1.00; 1.06) |
| Pal Choudhury | 2020 | GBR | iCARE-Lit |  | Light | Lit | 1.03 | NA | 1.03 |
| Pal Choudhury | 2020 | GBR | iCARE-BPC3 |  | Light | LR | 0.99 (0.92;1.07) | 0.01 | 0.99 (0.92;1.07) |
| Hüsing | 2012 | GBR | Covariate M |  | Extremes | LR | 1.32 (1.16;1.51) | 0.43 | 1.16 (1.08;1.24) |
| Hippisley-Cox | 2015 | GBR | Qcancer |  | Extremes | CPhM | 1.25 (0.92;1.71) | 0.02 | 1.25 (0.92;1.70) |
| Maas | 2016 | EUR. AUS. USA | NM-MRFS |  | Extremes | LR | 1.24 (1.14;1.34) | 0.46 | 1.12 (1.07;1.16) |
| Dierssen-Sotos | 2018 | ESP | MRFS |  | Extremes | LR | 1.01 (0.74;1.36) | 0.47 | 1.01 (0.84;1.16) |
| Li | 2018 | EPIC | ER+/ER-/Omnibus | | Extremes | CPhM | 1.22 (1.12;1.33) | 0.00 | 1.22 (1.12;1.33) |
| Rudolph | 2018 | EUR. AUS. USA | NM-MRFs |  | Extremes | LR | 1.35 (0.93;1.95) | 0.49 | 1.15 (0.96;1.33) |
| Lee | 2019 | GBR | Ext BOADICEA | | Extremes | Lit | 1.46 (1.30; 1.64) | NA | 1.46 (1.30; 1.64) |
| Pal Choudhury | 2020 | GBR | iCARE-Lit |  | Extremes | Lit | 1.46 | NA | 1.46 |
| Pal Choudhury | 2020 | GBR | iCARE-BPC3 |  | Extremes | LR | 1.22 (1.13;1.33) | 0.01 | 1.22 (1.13;1.33) |
| Colditz | 2000 | USA | YourCancerRisk | | Categorical | Lit | 1.40 | NA | 1.40 |
| Petracci | 2011 | ITA | 5NMRF+2MRF | | Categorical | LR | 1.27 (1.12;1.43) | 0.47 | 1.13 (1.06;1.19) |
| Petracci | 2011 | ITA | 5NMRF+2MRF | | Categorical | LR | 1.23 (0.95;1.59) | 0.47 | 1.11 (0.97;1.25) |
| Lophatananon | 2017 | GBR | YourCancerRisk UK | | Categorical | NS | 1.10 (1.00;1.20) | NA | 1.10 (1.00;1.20) |
| Eriksson | 2020 | SWE | KARMA+RF+PRS | PrM | Categorical | LR | 1.14 (0.82;1.59) | 0.07 | 1.13 (0.83;1.53) |
| Eriksson | 2020 | SWE | KARMA+RF+PRS | PoM | Categorical | LR | 1.17 (0.93;1.47) | 0.13 | 1.14 (0.94;1.38) |
| Boyle | 2004 | ITA | M-Nutrient | 50- | Ordinal | LR | 1.35 (1.19;1.53) | 0.45 | 1.17 (1.10;1.23) |
| Boyle | 2004 | ITA | M-Nutrient | 50+ | Ordinal | LR | 1.05 (0.97;1.15) | 0.47 | 1.03 (0.98;1.07) |
| Boyle | 2004 | ITA | M-Food | 50- | Ordinal | LR | 1.36 (1.20;1.55) | 0.45 | 1.17 (1.10;1.24) |
| Boyle | 2004 | ITA | M-Food | 50+ | Ordinal | LR | 1.05 (0.96;1.15) | NA | 0.00 |
| Pfeiffer | 2013 | USA | BCRmod | 50+ | Ordinal | LR | 1.06 (1.00;1.12) | 0.00 | 1.06 (1.00;1.12) |
| Hüsing | 2020 | DEU | BCRmod | 50+ | Ordinal | LR | 1.14 (0.93;1.39) | 0.03 | 1.14 (0.93;1.38) |
| Usher-Smith | 2019 | GBR |  |  | Continuous | Lit | 1.07 (1.05;1.10) | NA | 1.07 (1.05;1.10) |

D: Physical activity as predictor

| **Author** | **Year** | **Country** | **ModelName** | **Population** | | **Comparison** | **Model** | **Published ES** | **PoRo** | **Transformed RR** |
| --- | --- | --- | --- | --- | --- | --- | --- | --- | --- | --- |
| Colditz | 2000 | USA | YourCancerRisk | | | Categorical | Lit | 0.80 | NA | 0.80 |
| Park | 2013 | KOR | KoBCRAT | | 50- | Categorical | LR | 0.75 (0.63;0.90) | 0.65 | 0.90 (0.83;0.96) |
| Park | 2013 | KOR | KoBCRAT | | 50+ | Categorical | LR | 0.54 (0.44;0.67) | 0.65 | 0.77 (0.69;0.85) |
| Lophatananon | 2017 | GBR | YourCancerRisk UK | | | Categorical | NS | 0.90 (0.80;0.90) | NA | 0.90 (0.80;0.90) |
| Usher-Smith | 2019 | GBR |  | |  | Continuous | Lit | 0.97 (0.95;0.98) | NA | 0.97 (0.95;0.98) |
| Petracci | 2011 | ITA | 5NMRF + 2MRF | | | Ordinal | LR | 0.92 (0.82;1.05) | 0.51 | 0.96 (0.90;1.02) |
| Boyle | 2004 | ITA | M-Nutrient | | 50+ | Ordinal | LR | 0.85 (0.74;0.99) | 0.59 | 0.93 (0.87;1.00) |
| Boyle | 2004 | ITA | M-Food | | 50+ | Ordinal | LR | 0.87 (0.75;1.00) | 0.59 | 0.94 (0.88;1.00) |
| Petracci | 2011 | ITA | 5NMRF + 2MRF | | | Ordinal | LR | 0.91 (0.81;1.03) | 0.61 | 0.96 (0.91;1.01) |

E: Smoking status as predictor

| **Author** | **Year** | **Country** | **ModelName** | **Population** | **Comparison** | **Model** | **Published ES** | **PoRo** | **Transformed RR** |
| --- | --- | --- | --- | --- | --- | --- | --- | --- | --- |
| Gabrielson | 2018 | SWE |  | PoM | Categorical | LR | 1.34 | 0.32 | 1.21 |
| Eriksson | 2020 | SWE |  | PrM | Categorical | LR | 0.82 (0.56;1.22) | 0.07 | 0.83 (0.58;1.20) |
| Eriksson | 2020 | SWE |  | PoM | Categorical | LR | 1.17 (0.90;1.51) | 0.13 | 1.14 (0.91;1.41) |
| Hüsing | 2012 | GBR | Covariate M |  | Categorical | LR | 1.09 (0.95;1.24) | 0.43 | 1.05 (0.97;1.12) |
| Pal Choudhury | 2020 | GBR | iCARE-BPC3 |  | Categorical | LR | 1.09 (1.04;1.13) | 0.01 | 1.09 (1.04;1.13) |
| Yiangou | 2021 | CYP |  |  | Categorical | LR | 1.18 (0.92;1.52) | 0.48 | 1.09 (0.96;1.22) |

*Abbreviations:* CPhM, Cox Proportional hazard Models; ES, effect size; Lit, literature-based; LR, logistic regression; NA, not applicable; PoRo, incidence proportion of outcome in non-exposed or incidence rate of outcome in the non-exposed; RR, relative risk.

References

[1] E. Amir, D.G. Evans, A. Shenton, F. Lalloo, A. Moran, C. Boggis, M. Wilson, and A. Howell, Evaluation of breast cancer risk assessment packages in the family history evaluation and screening programme. J Med Genet 40 (2003) 807-14.

[2] J. Tyrer, S.W. Duffy, and J. Cuzick, A breast cancer prediction model incorporating familial and personal risk factors. Stat Med 23 (2004) 1111-30.

[3] P. Boyle, M. Mezzetti, C. La Vecchia, S. Franceschi, A. Decarli, and C. Robertson, Contribution of three components to individual cancer risk predicting breast cancer risk in Italy. Eur J Cancer Prev 13 (2004) 183-91.

[4] A. Decarli, S. Calza, G. Masala, C. Specchia, D. Palli, and M.H. Gail, Gail model for prediction of absolute risk of invasive breast cancer: independent evaluation in the Florence-European Prospective Investigation Into Cancer and Nutrition cohort. J Natl Cancer Inst 98 (2006) 1686-93.

[5] J. Novotny, L. Pecen, L. Petruzelka, A. Svobodnik, L. Dusek, J. Danes, and M. Skovajsova, Breast cancer risk assessment in the Czech female population--an adjustment of the original Gail model. Breast Cancer Res Treat 95 (2006) 29-35.

[6] A. Crispo, G. D'Aiuto, M. De Marco, M. Rinaldo, M. Grimaldi, I. Capasso, A. Amore, C. Bosetti, C. La Vecchia, and M. Montella, Gail model risk factors: impact of adding an extended family history for breast cancer. Breast J 14 (2008) 221-7.

[7] V. Viallon, S. Ragusa, F. Clavel-Chapelon, and J. Bénichou, How to evaluate the calibration of a disease risk prediction tool. Stat Med 28 (2009) 901-16.

[8] C. Ulusoy, I. Kepenekci, K. Kose, S. Aydintug, and R. Cam, Applicability of the Gail model for breast cancer risk assessment in Turkish female population and evaluation of breastfeeding as a risk factor. Breast Cancer Res Treat 120 (2010) 419-24.

[9] S. Wacholder, P. Hartge, R. Prentice, M. Garcia-Closas, H.S. Feigelson, W.R. Diver, M.J. Thun, D.G. Cox, S.E. Hankinson, P. Kraft, B. Rosner, C.D. Berg, L.A. Brinton, J. Lissowska, M.E. Sherman, R. Chlebowski, C. Kooperberg, R.D. Jackson, D.W. Buckman, P. Hui, R. Pfeiffer, K.B. Jacobs, G.D. Thomas, R.N. Hoover, M.H. Gail, S.J. Chanock, and D.J. Hunter, Performance of common genetic variants in breast-cancer risk models. N Engl J Med 362 (2010) 986-93.

[10] E. Petracci, A. Decarli, C. Schairer, R.M. Pfeiffer, D. Pee, G. Masala, D. Palli, and M.H. Gail, Risk factor modification and projections of absolute breast cancer risk. J Natl Cancer Inst 103 (2011) 1037-48.

[11] H. Darabi, K. Czene, W. Zhao, J. Liu, P. Hall, and K. Humphreys, Breast cancer risk prediction and individualised screening based on common genetic variation and breast density measurement. Breast Cancer Res 14 (2012) R25.

[12] A. Hüsing, F. Canzian, L. Beckmann, M. Garcia-Closas, W.R. Diver, M.J. Thun, C.D. Berg, R.N. Hoover, R.G. Ziegler, J.D. Figueroa, C. Isaacs, A. Olsen, V. Viallon, H. Boeing, G. Masala, D. Trichopoulos, P.H. Peeters, E. Lund, E. Ardanaz, K.T. Khaw, P. Lenner, L.N. Kolonel, D.O. Stram, L. Le Marchand, C.A. McCarty, J.E. Buring, I.M. Lee, S. Zhang, S. Lindström, S.E. Hankinson, E. Riboli, D.J. Hunter, B.E. Henderson, S.J. Chanock, C.A. Haiman, P. Kraft, and R. Kaaks, Prediction of breast cancer risk by genetic risk factors, overall and by hormone receptor status. J Med Genet 49 (2012) 601-8.

[13] C. Rauh, C.C. Hack, L. Häberle, A. Hein, A. Engel, M.G. Schrauder, P.A. Fasching, S.M. Jud, A.B. Ekici, C.R. Loehberg, M. Meier-Meitinger, S. Ozan, R. Schulz-Wendtland, M. Uder, A. Hartmann, D.L. Wachter, M.W. Beckmann, and K. Heusinger, Percent Mammographic Density and Dense Area as Risk Factors for Breast Cancer. Geburtshilfe Frauenheilkd 72 (2012) 727-733.

[14] A. Arrospide, C. Forné, M. Rué, N. Torà, J. Mar, and M. Baré, An assessment of existing models for individualized breast cancer risk estimation in a screening program in Spain. BMC Cancer 13 (2013) 587.

[15] A. Buron, M. Vernet, M. Roman, M.A. Checa, J.M. Pérez, M. Sala, M. Comas, C. Murta-Nascimiento, X. Castells, and F. Macià, Can the Gail model increase the predictive value of a positive mammogram in a European population screening setting? Results from a Spanish cohort. Breast 22 (2013) 83-8.

[16] R. Pastor-Barriuso, N. Ascunce, M. Ederra, N. Erdozáin, A. Murillo, J.E. Alés-Martínez, and M. Pollán, Recalibration of the Gail model for predicting invasive breast cancer risk in Spanish women: a population-based cohort study. Breast Cancer Res Treat 138 (2013) 249-59.

[17] J. Warwick, H. Birke, J. Stone, R.M.L. Warren, E. Pinney, A.R. Brentnall, S.W. Duffy, A. Howell, and J. Cuzick, Mammographic breast density refines Tyrer-Cuzick estimates of breast cancer risk in high-risk women: findings from the placebo arm of the International Breast Cancer Intervention Study I. Breast Cancer Research 16 (2014) 451.

[18] A.R. Brentnall, E.F. Harkness, S.M. Astley, L.S. Donnelly, P. Stavrinos, S. Sampson, L. Fox, J.C. Sergeant, M.N. Harvie, M. Wilson, U. Beetles, S. Gadde, Y. Lim, A. Jain, S. Bundred, N. Barr, V. Reece, A. Howell, J. Cuzick, and D.G. Evans, Mammographic density adds accuracy to both the Tyrer-Cuzick and Gail breast cancer risk models in a prospective UK screening cohort. Breast Cancer Res 17 (2015) 147.

[19] L. Dartois, É. Gauthier, J. Heitzmann, L. Baglietto, S. Michiels, S. Mesrine, M.C. Boutron-Ruault, S. Delaloge, S. Ragusa, F. Clavel-Chapelon, and G. Fagherazzi, A comparison between different prediction models for invasive breast cancer occurrence in the French E3N cohort. Breast Cancer Res Treat 150 (2015) 415-26.

[20] J. Hippisley-Cox, and C. Coupland, Development and validation of risk prediction algorithms to estimate future risk of common cancers in men and women: prospective cohort study. BMJ Open 5 (2015) e007825.

[21] C.M. Vachon, V.S. Pankratz, C.G. Scott, L. Haeberle, E. Ziv, M.R. Jensen, K.R. Brandt, D.H. Whaley, J.E. Olson, K. Heusinger, C.C. Hack, S.M. Jud, M.W. Beckmann, R. Schulz-Wendtland, J.A. Tice, A.D. Norman, J.M. Cunningham, K.S. Purrington, D.F. Easton, T.A. Sellers, K. Kerlikowske, P.A. Fasching, and F.J. Couch, The contributions of breast density and common genetic variation to breast cancer risk. J Natl Cancer Inst 107 (2015).

[22] P. Maas, M. Barrdahl, A.D. Joshi, P.L. Auer, M.M. Gaudet, R.L. Milne, F.R. Schumacher, W.F. Anderson, D. Check, S. Chattopadhyay, L. Baglietto, C.D. Berg, S.J. Chanock, D.G. Cox, J.D. Figueroa, M.H. Gail, B.I. Graubard, C.A. Haiman, S.E. Hankinson, R.N. Hoover, C. Isaacs, L.N. Kolonel, L. Le Marchand, I.M. Lee, S. Lindström, K. Overvad, I. Romieu, M.J. Sanchez, M.C. Southey, D.O. Stram, R. Tumino, T.J. VanderWeele, W.C. Willett, S. Zhang, J.E. Buring, F. Canzian, S.M. Gapstur, B.E. Henderson, D.J. Hunter, G.G. Giles, R.L. Prentice, R.G. Ziegler, P. Kraft, M. Garcia-Closas, and N. Chatterjee, Breast Cancer Risk From Modifiable and Nonmodifiable Risk Factors Among White Women in the United States. JAMA Oncol 2 (2016) 1295-1302.

[23] J. Cuzick, A.R. Brentnall, C. Segal, H. Byers, C. Reuter, S. Detre, E. Lopez-Knowles, I. Sestak, A. Howell, T.J. Powles, W.G. Newman, and M. Dowsett, Impact of a Panel of 88 Single Nucleotide Polymorphisms on the Risk of Breast Cancer in High-Risk Women: Results From Two Randomized Tamoxifen Prevention Trials. Journal of Clinical Oncology 35 (2017) 743-750.

[24] M. Eriksson, K. Czene, Y. Pawitan, K. Leifland, H. Darabi, and P. Hall, A clinical model for identifying the short-term risk of breast cancer. Breast Cancer Res 19 (2017) 29.

[25] D. Evans, A.R. Brentnall, H. Byers, E. Harkness, P. Stavrinos, A. Howell, W. Newman, and J. Cuzick, Use of multiple Single Nucleotide Polymorphism (SNP) testing to predict breast cancer risk in a familial screening clinic. Journal of Medical Genetics (2016).

[26] A. Hüsing, R.T. Fortner, T. Kühn, K. Overvad, A. Tjønneland, A. Olsen, M.C. Boutron-Ruault, G. Severi, A. Fournier, H. Boeing, A. Trichopoulou, V. Benetou, P. Orfanos, G. Masala, V. Pala, R. Tumino, F. Fasanelli, S. Panico, H.B. Bueno de Mesquita, P.H. Peeters, C.H. van Gills, J.R. Quirós, A. Agudo, M.J. Sánchez, M.D. Chirlaque, A. Barricarte, P. Amiano, K.T. Khaw, R.C. Travis, L. Dossus, K. Li, P. Ferrari, M.A. Merritt, I. Tzoulaki, E. Riboli, and R. Kaaks, Added Value of Serum Hormone Measurements in Risk Prediction Models for Breast Cancer for Women Not Using Exogenous Hormones: Results from the EPIC Cohort. Clin Cancer Res 23 (2017) 4181-4189.

[27] A. Lophatananon, J. Usher-Smith, J. Campbell, J. Warcaba, B. Silarova, E.A. Waters, G.A. Colditz, and K.R. Muir, Development of a Cancer Risk Prediction Tool for Use in the UK Primary Care and Community Settings. Cancer Prev Res (Phila) 10 (2017) 421-430.

[28] T. Dierssen-Sotos, I. Gómez-Acebo, C. Palazuelos, P. Fernández-Navarro, J.M. Altzibar, C. González-Donquiles, E. Ardanaz, M. Bustamante, J. Alonso-Molero, C. Vidal, J. Bayo-Calero, A. Tardón, D. Salas, R. Marcos-Gragera, V. Moreno, P. Rodriguez-Cundin, G. Castaño-Vinyals, M. Ederra, L. Vilorio-Marqués, P. Amiano, B. Pérez-Gómez, N. Aragonés, M. Kogevinas, M. Pollán, and J. Llorca, Validating a breast cancer score in Spanish women. The MCC-Spain study. Scientific Reports 8 (2018) 3036.

[29] M. Gabrielson, K. Ubhayasekera, B. Ek, M. Andersson Franko, M. Eriksson, K. Czene, J. Bergquist, and P. Hall, Inclusion of Plasma Prolactin Levels in Current Risk Prediction Models of Premenopausal and Postmenopausal Breast Cancer. JNCI Cancer Spectr 2 (2018) pky055.

[30] K. Li, G. Anderson, V. Viallon, P. Arveux, M. Kvaskoff, A. Fournier, V. Krogh, R. Tumino, M.-J. Sánchez, E. Ardanaz, M.-D. Chirlaque, A. Agudo, D.C. Muller, T. Smith, I. Tzoulaki, T.J. Key, B. Bueno-de-Mesquita, A. Trichopoulou, C. Bamia, P. Orfanos, R. Kaaks, A. Hüsing, R.T. Fortner, A. Zeleniuch-Jacquotte, M. Sund, C.C. Dahm, K. Overvad, D. Aune, E. Weiderpass, I. Romieu, E. Riboli, M.J. Gunter, L. Dossus, R. Prentice, and P. Ferrari, Risk prediction for estrogen receptor-specific breast cancers in two large prospective cohorts. Breast Cancer Research 20 (2018) 147.

[31] F. Lumachi, S.M.M. Basso, V. Camozzi, R. Spaziante, P. Ubiali, and M. Ermani, Bone Mineral Density as a Potential Predictive Factor for Luminal-type Breast Cancer in Postmenopausal Women. Anticancer Res 38 (2018) 3049-3054.

[32] A. Rudolph, M. Song, M.N. Brook, R.L. Milne, N. Mavaddat, K. Michailidou, M.K. Bolla, Q. Wang, J. Dennis, A.N. Wilcox, J.L. Hopper, M.C. Southey, R. Keeman, P.A. Fasching, M.W. Beckmann, M. Gago-Dominguez, J.E. Castelao, P. Guénel, T. Truong, S.E. Bojesen, H. Flyger, H. Brenner, V. Arndt, H. Brauch, T. Brüning, A. Mannermaa, V.M. Kosma, D. Lambrechts, M. Keupers, F.J. Couch, C. Vachon, G.G. Giles, R.J. MacInnis, J. Figueroa, L. Brinton, K. Czene, J.S. Brand, M. Gabrielson, K. Humphreys, A. Cox, S.S. Cross, A.M. Dunning, N. Orr, A. Swerdlow, P. Hall, P.D.P. Pharoah, M.K. Schmidt, D.F. Easton, N. Chatterjee, J. Chang-Claude, and M. García-Closas, Joint associations of a polygenic risk score and environmental risk factors for breast cancer in the Breast Cancer Association Consortium. Int J Epidemiol 47 (2018) 526-536.

[33] E.M. van Veen, A.R. Brentnall, H. Byers, E.F. Harkness, S.M. Astley, S. Sampson, A. Howell, W.G. Newman, J. Cuzick, and D.G.R. Evans, Use of Single-Nucleotide Polymorphisms and Mammographic Density Plus Classic Risk Factors for Breast Cancer Risk Prediction. JAMA Oncol 4 (2018) 476-482.

[34] T.V. Clendenen, W. Ge, K.L. Koenig, Y. Afanasyeva, C. Agnoli, L.A. Brinton, F. Darvishian, J.F. Dorgan, A.H. Eliassen, R.T. Falk, G. Hallmans, S.E. Hankinson, J. Hoffman-Bolton, T.J. Key, V. Krogh, H.B. Nichols, D.P. Sandler, M.J. Schoemaker, P.M. Sluss, M. Sund, A.J. Swerdlow, K. Visvanathan, A. Zeleniuch-Jacquotte, and M. Liu, Breast cancer risk prediction in women aged 35–50 years: impact of including sex hormone concentrations in the Gail model. Breast Cancer Research 21 (2019) 42.

[35] A. Lee, N. Mavaddat, A.N. Wilcox, A.P. Cunningham, T. Carver, S. Hartley, C. Babb de Villiers, A. Izquierdo, J. Simard, M.K. Schmidt, F.M. Walter, N. Chatterjee, M. Garcia-Closas, M. Tischkowitz, P. Pharoah, D.F. Easton, and A.C. Antoniou, BOADICEA: a comprehensive breast cancer risk prediction model incorporating genetic and nongenetic risk factors. Genet Med 21 (2019) 1708-1718.

[36] J.A. Usher-Smith, S.J. Sharp, R. Luben, and S.J. Griffin, Development and Validation of Lifestyle-Based Models to Predict Incidence of the Most Common Potentially Preventable Cancers. Cancer Epidemiol Biomarkers Prev 28 (2019) 67-75.

[37] A.R. Brentnall, E.M. van Veen, E.F. Harkness, S. Rafiq, H. Byers, S.M. Astley, S. Sampson, A. Howell, W.G. Newman, J. Cuzick, and D.G.R. Evans, A case-control evaluation of 143 single nucleotide polymorphisms for breast cancer risk stratification with classical factors and mammographic density. Int J Cancer 146 (2020) 2122-2129.

[38] P. Pal Choudhury, A.N. Wilcox, M.N. Brook, Y. Zhang, T. Ahearn, N. Orr, P. Coulson, M.J. Schoemaker, M.E. Jones, M.H. Gail, A.J. Swerdlow, N. Chatterjee, and M. Garcia-Closas, Comparative Validation of Breast Cancer Risk Prediction Models and Projections for Future Risk Stratification. J Natl Cancer Inst 112 (2020) 278-285.

[39] M. Eriksson, K. Czene, F. Strand, S. Zackrisson, P. Lindholm, K. Lång, D. Förnvik, H. Sartor, N. Mavaddat, D. Easton, and P. Hall, Identification of Women at High Risk of Breast Cancer Who Need Supplemental Screening. Radiology 297 (2020) 327-333.

[40] M. Gabrielson, K.A. Ubhayasekera, S.R. Acharya, M.A. Franko, M. Eriksson, J. Bergquist, K. Czene, and P. Hall, Inclusion of Endogenous Plasma Dehydroepiandrosterone Sulfate and Mammographic Density in Risk Prediction Models for Breast Cancer. Cancer Epidemiol Biomarkers Prev 29 (2020) 574-581.

[41] Z. Guan, J.R. Raut, K. Weigl, B. Schöttker, B. Holleczek, Y. Zhang, and H. Brenner, Individual and joint performance of DNA methylation profiles, genetic risk score and environmental risk scores for predicting breast cancer risk. Mol Oncol 14 (2020) 42-53.

[42] A. Hüsing, A.S. Quante, J. Chang-Claude, K. Aleksandrova, R. Kaaks, and R.M. Pfeiffer, Validation of two US breast cancer risk prediction models in German women. Cancer Causes Control 31 (2020) 525-536.

[43] I.M.M. Lakeman, M. Rodríguez-Girondo, A. Lee, R. Ruiter, B.H. Stricker, S.R.A. Wijnant, M. Kavousi, A.C. Antoniou, M.K. Schmidt, A.G. Uitterlinden, J. van Rooij, and P. Devilee, Validation of the BOADICEA model and a 313-variant polygenic risk score for breast cancer risk prediction in a Dutch prospective cohort. Genet Med 22 (2020) 1803-1811.

[44] J.C. Triviño, A. Ceba, E. Rubio-Solsona, D. Serra, I. Sanchez-Guiu, G. Ribas, R. Rosa, M. Cabo, L. Bernad, G. Pita, A. Gonzalez-Neira, G. Legarda, J.L. Diaz, A. García-Vigara, A. Martínez-Aspas, M. Escrig, B. Bermejo, P. Eroles, J. Ibáñez, D. Salas, A. Julve, A. Cano, A. Lluch, R. Miñambres, and J. Benitez, Combination of phenotype and polygenic risk score in breast cancer risk evaluation in the Spanish population: a case -control study. BMC Cancer 20 (2020) 1079.

[45] E. Bonnet, J.P. Daures, and P. Landais, Determination of thresholds of risk in women at average risk of breast cancer to personalize the organized screening program. Sci Rep 11 (2021) 19104.

[46] P. Pal Choudhury, M.N. Brook, A.N. Hurson, A. Lee, C.V. Mulder, P. Coulson, M.J. Schoemaker, M.E. Jones, A.J. Swerdlow, N. Chatterjee, A.C. Antoniou, and M. Garcia-Closas, Comparative validation of the BOADICEA and Tyrer-Cuzick breast cancer risk models incorporating classical risk factors and polygenic risk in a population-based prospective cohort of women of European ancestry. Breast Cancer Res 23 (2021) 22.

[47] A.N. Hurson, P. Pal Choudhury, C. Gao, A. Hüsing, M. Eriksson, M. Shi, M.E. Jones, D.G.R. Evans, R.L. Milne, M.M. Gaudet, C.M. Vachon, D.I. Chasman, D.F. Easton, M.K. Schmidt, P. Kraft, M. Garcia-Closas, and N. Chatterjee, Prospective evaluation of a breast-cancer risk model integrating classical risk factors and polygenic risk in 15 cohorts from six countries. Int J Epidemiol 50 (2022) 1897-1911.

[48] J. Louro, M. Román, M. Posso, I. Vázquez, F. Saladié, A. Rodriguez-Arana, M.J. Quintana, L. Domingo, M. Baré, R. Marcos-Gragera, M. Vernet-Tomas, M. Sala, and X. Castells, Developing and validating an individualized breast cancer risk prediction model for women attending breast cancer screening. PLoS One 16 (2021) e0248930.

[49] K. Yiangou, K. Kyriacou, E. Kakouri, Y. Marcou, M.I. Panayiotidis, M.A. Loizidou, A. Hadjisavvas, and K. Michailidou, Combination of a 15-SNP Polygenic Risk Score and Classical Risk Factors for the Prediction of Breast Cancer Risk in Cypriot Women. Cancers (Basel) 13 (2021).

[50] A.C. Antoniou, A.P. Cunningham, J. Peto, D.G. Evans, F. Lalloo, S.A. Narod, H.A. Risch, J.E. Eyfjord, J.L. Hopper, M.C. Southey, H. Olsson, O. Johannsson, A. Borg, B. Pasini, P. Radice, S. Manoukian, D.M. Eccles, N. Tang, E. Olah, H. Anton-Culver, E. Warner, J. Lubinski, J. Gronwald, B. Gorski, L. Tryggvadottir, K. Syrjakoski, O.P. Kallioniemi, H. Eerola, H. Nevanlinna, P.D. Pharoah, and D.F. Easton, The BOADICEA model of genetic susceptibility to breast and ovarian cancers: updates and extensions. Br J Cancer 98 (2008) 1457-66.

[51] M.H. Gail, L.A. Brinton, D.P. Byar, D.K. Corle, S.B. Green, C. Schairer, and J.J. Mulvihill, Projecting individualized probabilities of developing breast cancer for white females who are being examined annually. J Natl Cancer Inst 81 (1989) 1879-86.

[52] M.H. Gail, and J.P. Costantino, Validating and Improving Models for Projecting the Absolute Risk of Breast Cancer. JNCI: Journal of the National Cancer Institute 93 (2001) 334-335.

[53] J.P. Costantino, M.H. Gail, D. Pee, S. Anderson, C.K. Redmond, J. Benichou, and H.S. Wieand, Validation studies for models projecting the risk of invasive and total breast cancer incidence. J Natl Cancer Inst 91 (1999) 1541-8.

[54] M.H. Gail, J.P. Costantino, D. Pee, M. Bondy, L. Newman, M. Selvan, G.L. Anderson, K.E. Malone, P.A. Marchbanks, W. McCaskill-Stevens, S.A. Norman, M.S. Simon, R. Spirtas, G. Ursin, and L. Bernstein, Projecting individualized absolute invasive breast cancer risk in African American women. J Natl Cancer Inst 99 (2007) 1782-92.

[55] R.K. Matsuno, J.P. Costantino, R.G. Ziegler, G.L. Anderson, H. Li, D. Pee, and M.H. Gail, Projecting individualized absolute invasive breast cancer risk in Asian and Pacific Islander American women. J Natl Cancer Inst 103 (2011) 951-61.

[56] M.P. Banegas, E.M. John, M.L. Slattery, S.L. Gomez, M. Yu, A.Z. LaCroix, D. Pee, R.T. Chlebowski, L.M. Hines, C.A. Thompson, and M.H. Gail, Projecting Individualized Absolute Invasive Breast Cancer Risk in US Hispanic Women. J Natl Cancer Inst 109 (2017).

[57] R.M. Pfeiffer, Y. Park, A.R. Kreimer, J.V. Lacey, Jr., D. Pee, R.T. Greenlee, S.S. Buys, A. Hollenbeck, B. Rosner, M.H. Gail, and P. Hartge, Risk prediction for breast, endometrial, and ovarian cancer in white women aged 50 y or older: derivation and validation from population-based cohort studies. PLoS Med 10 (2013) e1001492.

[58] B. Rosner, and G.A. Colditz, Nurses' health study: log-incidence mathematical model of breast cancer incidence. J Natl Cancer Inst 88 (1996) 359-64.

[59] G.A. Colditz, and B. Rosner, Cumulative risk of breast cancer to age 70 years according to risk factor status: data from the Nurses' Health Study. Am J Epidemiol 152 (2000) 950-64.

[60] G.A. Colditz, K.A. Atwood, K. Emmons, R.R. Monson, W.C. Willett, D. Trichopoulos, and D.J. Hunter, Harvard report on cancer prevention volume 4: Harvard Cancer Risk Index. Risk Index Working Group, Harvard Center for Cancer Prevention. Cancer Causes Control 11 (2000) 477-88.

[61] Siteman Cancer Center at Barnes-Jewish Hospital and Washington University School of Medicine, Your Disease Risk, Barnes-Jewish Hospital and Washington University School of Medicine, 2017.

[62] W.E. Barlow, E. White, R. Ballard-Barbash, P.M. Vacek, L. Titus-Ernstoff, P.A. Carney, J.A. Tice, D.S. Buist, B.M. Geller, R. Rosenberg, B.C. Yankaskas, and K. Kerlikowske, Prospective breast cancer risk prediction model for women undergoing screening mammography. J Natl Cancer Inst 98 (2006) 1204-14.

[63] J. Chen, D. Pee, R. Ayyagari, B. Graubard, C. Schairer, C. Byrne, J. Benichou, and M.H. Gail, Projecting absolute invasive breast cancer risk in white women with a model that includes mammographic density. J Natl Cancer Inst 98 (2006) 1215-26.

[64] J.A. Tice, S.R. Cummings, R. Smith-Bindman, L. Ichikawa, W.E. Barlow, and K. Kerlikowske, Using clinical factors and mammographic breast density to estimate breast cancer risk: development and validation of a new predictive model. Ann Intern Med 148 (2008) 337-47.

[65] B. Park, S.H. Ma, A. Shin, M.C. Chang, J.Y. Choi, S. Kim, W. Han, D.Y. Noh, S.H. Ahn, D. Kang, K.Y. Yoo, and S.K. Park, Korean risk assessment model for breast cancer risk prediction. PLoS One 8 (2013) e76736.

[66] F. Wang, J. Dai, M. Li, W.C. Chan, C.C. Kwok, S.L. Leung, C. Wu, W. Li, W.C. Yu, K.H. Tsang, S.H. Law, P.M. Lee, C.K. Wong, H. Shen, S.Y. Wong, X.R. Yang, and L.A. Tse, Risk assessment model for invasive breast cancer in Hong Kong women. Medicine (Baltimore) 95 (2016) e4515.
